# Socioemotional and Executive Control Mismatch in Adolescence and Risks for Initiating Drinking

**DOI:** 10.1001/jamanetworkopen.2025.31378

**Published:** 2025-09-12

**Authors:** Qingyu Zhao, Leo Milecki, Amy Kuceyeski, Logan Grosenick, Ty Brumback, Adolf Pfefferbaum, Edith V. Sullivan, Kilian M. Pohl

**Affiliations:** 1 Department of Radiology, Weill Cornell Medicine, New York, New York; 2 Department of Psychiatry, Weill Cornell Medicine, New York, New York; 3 School of Psychology, Xavier University, Cincinnati, Ohio; 4 Center for Health Sciences, SRI International, Menlo Park, California; 5 Department of Psychiatry and Behavioral Sciences, Stanford University, Stanford, California; 6 AI Editor, *JAMA Psychiatry*

## Abstract

**Importance:**

Maturational imbalance between the expression of heightened socioemotional drivers and immature executive control creates a period of vulnerability to risk-taking behaviors during adolescence. Developmental imbalance of the brain functional activity coupled with these behaviors may explain the propensity to develop heavy drinking.

**Objective:**

To compare the longitudinal trajectories of socioemotion and executive control in non–heavy drinkers with those of heavy drinkers before and after drinking onset.

**Design, Setting, and Participants:**

This cohort study was conducted from January 13, 2013, to January 15, 2022, among participants from the National Consortium on Alcohol and Neurodevelopment in Adolescence cohort with resting-state functional magnetic resonance imaging and neuropsychological assessment data. All participants were individuals who abstained from drinking or drank very little at the baseline visit. The statistical analysis was conducted between October 11, 2024, and July 3, 2025.

**Exposures:**

Categorical drinking levels that summarized self-reported alcohol consumption in the past year.

**Main Outcomes and Measures:**

Brain-behavioral system *z* scores that reflected the association of selective brain connectivity patterns with selected behaviors were calculated. Higher scores indicated higher reactivity strength of socioemotion or executive control.

**Results:**

A total of 3076 visits among 633 participants (mean [SD] age at baseline, 15.7 [2.6] years; 318 female [50.2%]) were analyzed. A total of 238 participants (37.6%) who initiated heavy drinking during the study showed significantly elevated brain-behavior scores (score increase, 0.18; 95% CI, 0.08-0.28) in the socioemotional system prior to drinking onset, but their developmental trajectories of the executive control system followed those of the control participants. After drinking onset, developmental deviation occurred in both the socioemotional system (score increase, 0.44; 95% CI, 0.33-0.54) and executive control system (score increase, 0.21; 95% CI 0.11-0.32), with a significantly larger effect size in the socioemotional system (*z* = 3.51; *P* < .001) compared with before initiating drinking.

**Conclusions and Relevance:**

These findings suggest that asynchronized maturation of socioemotion- and executive function–coupled brain connectivity in adolescence may be a risk factor for heavy drinking onset in emerging adulthood. In turn, drinking was associated with deficits in the executive control system and exacerbated alteration in the socioemotional system. Recognition of these early-age developmental discrepancies could aid in averting alcohol use disorder in adulthood.

## Key Points

**Question:**

What is the association of imbalances between socioemotional and executive control brain-behavior systems in adolescence with risk for heavy alcohol drinking in emerging adulthood?

**Findings:**

In this cohort study of 633 participants aged 12 to 21 years at baseline, faster development of the socioemotional system was associated with drinking onset in individuals who initiated heavy drinking compared with those who refrained from initiating heavy drinking. Heavy drinking was associated with deviation in the executive control system and exacerbated socioemotional dysregulation.

**Meaning:**

These findings suggest that maturational imbalance may be a risk factor for heavy drinking onset, which in turn may pose risks for developing alcohol-induced damage of both brain-behavior systems.

## Teaser

This cohort study compares socioemotional and executive control brain-behavior systems in adolescent non–heavy alcohol drinkers vs heavy alcohol drinkers before and after drinking onset.

## Introduction

Adolescence is a period marked by emotionally driven choices and heightened impulsivity, often occurring before the full maturation of behavioral control mechanisms.^1,2^ This imbalance stems from brain-behavior systems that may develop at different rates. The socioemotional system becomes highly active and responsive to social rewards, peer influence, and emotional stimuli, often motivating impulsive actions.^3,4^ Its reactivity peaks in midadolescence and begins to stabilize in late adolescence to early adulthood as brain connectivity matures.^5^ Contemporaneously, the executive control system, which supports decision-making, planning, and impulse regulation,^6,7^ matures more gradually compared with the socioemotional system, with supportive brain structural and functional changes continuing substantially into the mid-20s.^8,9^ The asynchronized development of this dual system, that is, maturational imbalance, may be a cognitive mechanism that heightens risk taking,^2,5^ including heavy alcohol drinking, observed during adolescence and young adulthood.

Recent neuroimaging research has shed light on the effects of heavy alcohol consumption on the adolescent brain. Longitudinal structural magnetic resonance imaging (MRI) studies have revealed that heavy alcohol consumption during adolescence is associated with abnormally fast reductions of gray matter volume in regions such as the prefrontal cortex^10,11,12^ and hippocampus and with compromised microstructural integrity in the frontal commissural tracts.^13,14^ Functional imaging has shown altered neural activity in emotion,^15^ default-mode,^16^ and sensorimotor networks^17^ linked to impaired neuropsychological functions such as decision-making, impulse control, emotional dysregulation, and reward processing.^18,19,20,21^ Despite widely replicated neuroimaging findings, there remains an incomplete and incomprehensive understanding of how imbalanced neurodevelopment of the dual system contributes to drinking onset and how alcohol, in turn, dysregulates socioemotional function and dampens executive functional control that should otherwise provide protection against engagement in risky behavior. Addressing this lacuna requires identifying neural systems linked to the 2 behavior domains of socioemotion and executive control, estimating their concurrent developmental trajectories, and comparing normal trajectories with those of alcohol drinkers both before and after initiating heavy drinking.

Based on 9-year longitudinal resting-state functional MRI and neuropsychological testing data collected by the National Consortium on Alcohol and Neurodevelopment in Adolescence (NCANDA) project,^22^ the current study used a data-driven machine learning approach (eFigure 1 in Supplement 1) to discern developmental trajectories of the 2 brain-behavioral domains linked to socioemotion and executive control. By comparing trajectories among individuals who drink heavily (hereafter referred to as heavy drinkers) and those who do not (hereafter referred to as non–heavy drinkers) before and after drinking onset, we aimed to provide direct neurobiological evidence to support the conceptual hypothesis that heavy drinking behavior would emerge when socioemotional development outpaces the maturation of the executive control system. In turn, we investigated whether heavy drinking is associated with disrupted executive control functions and exacerbated socioemotional dysregulation.

## Methods

### Participants

This cohort study used NCANDA data from participants aged 12 to 21 years at baseline recruited across 5 collection sites in the US from January 13, 2013, to January 15, 2022, and assessed annually for psychobiological measures. The institutional review boards of each site approved data collection and use. Adult participants and parents of minor participants provided written informed consent before participation in the study. Minor participants provided assent before participation. This study followed the Strengthening the Reporting of Observational Studies in Epidemiology (STROBE) reporting guideline.

Based on self-reported alcohol use history released throughout the first 9 years of the study, drinking levels of participants were defined based on the youth-adjusted Cahalan score on a scale of 0 to 3.^10,23^ Heavy drinkers (Cahalan = 2 or 3) ranged from moderate frequency (eg, 2 times per month) with high-quantity consumption (eg, 3-4 drinks on average and >4 drinks maximum) to higher frequency (eg, ≥1 time per week) with moderate-quantity consumption (eg, 2-3 drinks on average and >4 drinks maximum). The current analysis focused on participants who abstained from drinking or drank very little (Cahalan = 0) at their baseline visits, had no brain structural anomaly,^24^ and had usable resting-state functional MRI scans during the study period. Given the right-tail skew of the age distribution (eFigure 2 in Supplement 1), we confined the maximum age to 26 years to avoid bias from the limited data points at older ages.

### MRI Data

The structural and resting-state brain data of all NCANDA participants were preprocessed using the publicly available NCANDA pipeline,^17^ which included motion correction, outlier detection, detrending, physiologic noise removal, and temporal and spatial smoothing. The pipeline produced the mean blood oxygen level–dependent signal from 109 brain regions defined by the SRI24 Atlas,^25^ including 53 bilateral regions and 3 cerebellum vermis regions. To reduce dimensionality, we computed a Pearson correlation coefficient of the blood oxygen level–dependent signal between each pair of regions and averaged the correlation values of the left and right hemispheres,^26^ resulting in 1378 functional connectivity measures for each participant visit. Following previous work,^13^ we adjusted the potential confounding effects of socioeconomic status (defined by the maximum years of education of either parent), data collection site, and self-reported race (Asian, Black, White, other [American Indian, Pacific Islander, multiracial]) from each connectivity measure by a linear mixed-effects regression and normalized the residualized measures to *z* scores. These variables were based on prior findings of the NCANDA dataset that they are associated with neuroimaging and neuropsychological data and may confound the analysis.^27,28^ Finally, we used principal component analysis to reduce the dimensionality from 1378 to 384 to preserve 90% of the variance in the data based on prior practice.^29,30^

### Neuropsychological Measurements

Measures were from 17 types of neuropsychological test batteries (eg, Penn Computerized Neurobehavioral Test Battery,^31^ delay discounting,^32^ grooved pegboard^33^) or behavioral questionnaires (eg, Urgency-Premeditation-Perseverance-Sensation Seeking-Positive Urgency Impulsive Behavior Scale,^34^ Behavior Rating Inventory of Executive Function,^35^ Alcohol Expectancy Questionnaire^36^). Each test battery or questionnaire was categorized into 1 of the 2 domains based on their primary intended use noted in the literature. For a test battery or a questionnaire that contained many subscale measures, we kept only a few summary scores to reduce the overall dimensionality (eg, of the 53 recorded subscales of the Stroop test,^37^ only the mean and SD of the response time over all correct trials were kept). Next, we removed measures that were missing for more than half of the participants and removed participant visits in which more than half of the neuropsychological measures were missing. This process resulted in 82 neuropsychological measures assigned to the executive functioning system and 44 measures assigned to the socioemotional system (eTables 1 and 2 in Supplement 1). For each measurement, a linear mixed-effects model regressed out socioeconomic status, data collection site, and race^13^ and normalized the residualized measures to *z* scores. Finally, missing data were imputed by a k-nearest neighbor imputation algorithm, as previously described.^19,38^

### Statistical Analysis

#### Canonical Correlation Analysis

A canonical correlation analysis (CCA)^29,39^ was conducted for each system separately (eFigure 1A in Supplement 1) to identify the high-dimensional covariate pattern between the 384 functional principal component analysis scores and neuropsychological measures across participant visits. The CCA was evaluated by a 10-fold participant-level cross validation. During training, CCA derived a set of canonical components, each represented by a pair of linear transformations of functional and neuropsychological measures that were highly correlated (a brain-behavioral mapping) (eFigure 1B in Supplement 1). The correlation value between the 2 transformed variables in each component was then computed on the testing fold and averaged over the 10 folds after cross validation. Statistical significance of the correlation values was determined by permutation tests and corrected by the number of neuropsychological measures in each system based on Bonferroni correction. The threshold for significance was set at *P* ≤ .05.

Next, we identified which individual neuropsychological measures contributed most to brain-behavior mapping. For a significant component, we averaged the canonical loading (eMethods in Supplement 1) of each neuropsychological measure over the 10 folds and used a permutation test to identify measures with significantly large canonical loadings. Finally, for each participant visit, the overall reactivity strength of a component (hereafter referred to as a brain-behavior score) was computed as the average between brain functional and neuropsychological canonical variables (ie, linear transformations of functional and neuropsychological measures) (eMethods in Supplement 1) after *z* score transformation.

#### Association Between Heavy Drinking and Dual-System Development

For each significant component, we compared the developmental trajectory of the brain-behavior score of non–heavy drinkers (Cahalan = 0 or 1) to the trajectory of heavy drinkers before and after drinking onset (eFigure 1B in Supplement 1). To examine whether there was developmental deviation predating drinking onset, a mixed-effects model with participant-specific intercepts was used to test the difference in brain-behavior scores between the 2 drinking groups, with age and sex as covariates. For the heavy drinkers, only participant visits prior to drinking onset were included in this regression. A stepwise approach^40^ was used to determine the highest-order polynomial term associated with age to account for the potential nonlinear developmental trajectory of a component. Statistical significance of the drinking effect was corrected by the number of significant components in CCA based on Bonferroni correction. Then, the same mixed-effects model was repeated by replacing the prior to drinking visits of the heavy drinkers with visits after drinking initiation. For each component showing a significant group difference, 3 exploratory analyses were performed. First, we added a sex-by-group or site-by-group interaction term to the mixed-effects model to test whether the group difference differed by sex or data collection site. Second, to test whether heavy drinking had a dose-response association with brain-behavior scores, the mixed-effects model was applied to heavy drinkers by replacing the drinking group variable with a continuous alcohol consumption variable (average number of drinks in the past month or number of binge drinking episodes in the past month). Third, we applied the mixed-effects model to test the alcohol effect on the trajectory of each individual neuropsychological measure with significant canonical loading. The statistical analysis was conducted between October 11, 2024, and July 3, 2025, using MATLAB, version R2022b (The MathWorks, Inc).

## Results

A total of 3076 visits from 633 participants (mean [SD] age at baseline, 15.7 [2.6] years; 318 female [50.2%] and 315 male [49.8%]; 60 self-reporting as Asian [9.5%], 80 as Black [12.6%], 466 as White [73.6%], and 37 as other [5.8%] race) were included in the analysis (Table). A total of 238 participants (37.6%) initiated heavy drinking before age 26 years. The remaining 395 participants (62.4%) who did not initiate heavy drinking were considered non–heavy drinkers.

**Table.** Demographics of NCANDA Participants During Follow-Up

Characteristic	Participants, No. (%)	
	Non–heavy drinkers (n = 395)	Heavy drinkers (n = 238)
Sex		
Female	217 (54.9)	101 (42.4)
Male	178 (45.1)	137 (57.6)
Age at baseline, mean (SD), y	15.7 (2.6)	16.0 (2.0)
No. of visits, mean (SD)	4.4 (1.7)	5.3 (1.6)
Family drinking history^a^	35 (12.8)	24 (12.4)
Race		
Asian	34 (8.6)	16 (6.7)
Black	64 (16.2)	16 (6.7)
White	273 (69.1)	193 (81.1)
Other^b^	24 (6.1)	13 (5.5)
Site		
University of Pittsburgh	62 (15.7)	25 (10.5)
SRI International	82 (20.7)	36 (15.1)
Duke	88 (22.3)	47 (19.7)
Oregon Health & Science University	71 (18.0)	57 (24.0)
University of California, San Diego	92 (23.3)	73 (30.7)
Socioeconomic status, mean (SD)^c^	16.6 (2.5)	17.1 (2.4)

^a^ Number of participants who had (1) at least 1 biological parent with substantial problems indicative of an alcohol disorder; (2) 2 or more biological grandparents with substantial problems indicative of an alcohol disorder; or (3) 1 or more biological grandparents and 2 or more other biological second-degree relatives (eg, aunt, uncle) with substantial problems indicative of an alcohol or other drug disorder.

^b^ Other race included American Indian, Pacific Islander, and multiracial.

^c^ Defined as maximum years of education of either parent.

### Socioemotional System

**Figure 1.**
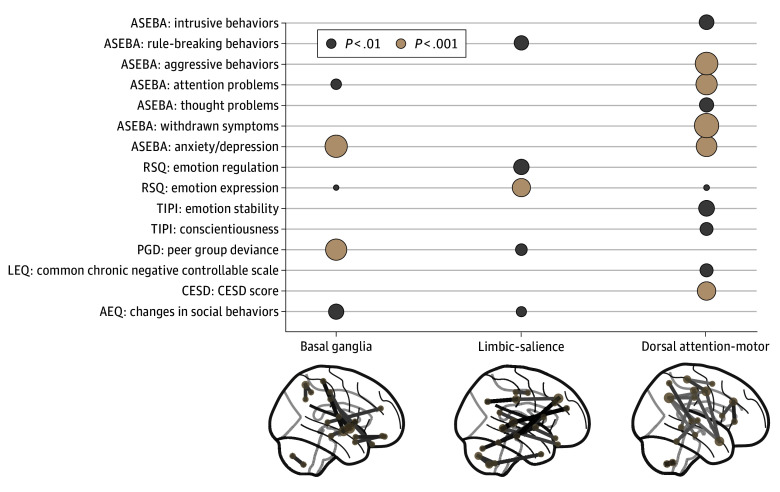
Significant Brain-Behavior Components in the Socioemotional Domain and Top 1% Functional Connectivity With Highest Canonical Loadings Each column displays the corresponding neuropsychological measures with significant loadings. Measures with nonsignificant loadings for all 3 components were omitted. AEQ indicates Alcohol Expectancy Questionnaire; ASEBA, Achenbach System of Empirically Based Assessment; CESD, The Center for Epidemiologic Studies Depression; LEQ, Life Event Questionnaire; PGD, peer group deviance; RSQ, Response to Stress Questionnaire; TIPI, Ten Item Personality Measure.

The CCA specific to the socioemotional system yielded 3 significant components (*P* < .001 by permutation test) (eFigure 3A in Supplement 1). These components were consistently identified based on different data processing methods (eFigures 4 and 5 in Supplement 1). Each component encoded a pattern of brain-behavior coupling between a functional brain network and a constellation of neuropsychological measures (Figure 1). Based on functional connectivities and neuropsychological measures with high canonical loadings (Figure 1; eTable 3 in Supplement 1), the first socioemotional component showed an association between functional connectivity in basal ganglia and symptoms of anxiety and depression and peer group deviance score. The second component showed an association between functional connectivity in limbic regions and the salience network and subscales of the Response to Stress Questionnaire.^41^ The third component showed an association between functional connectivity in dorsal attention and motor networks and subscales of the Childhood Behavior Checklist defined by the Achenbach System of Empirically Based Assessment.^42^

**Figure 2.**
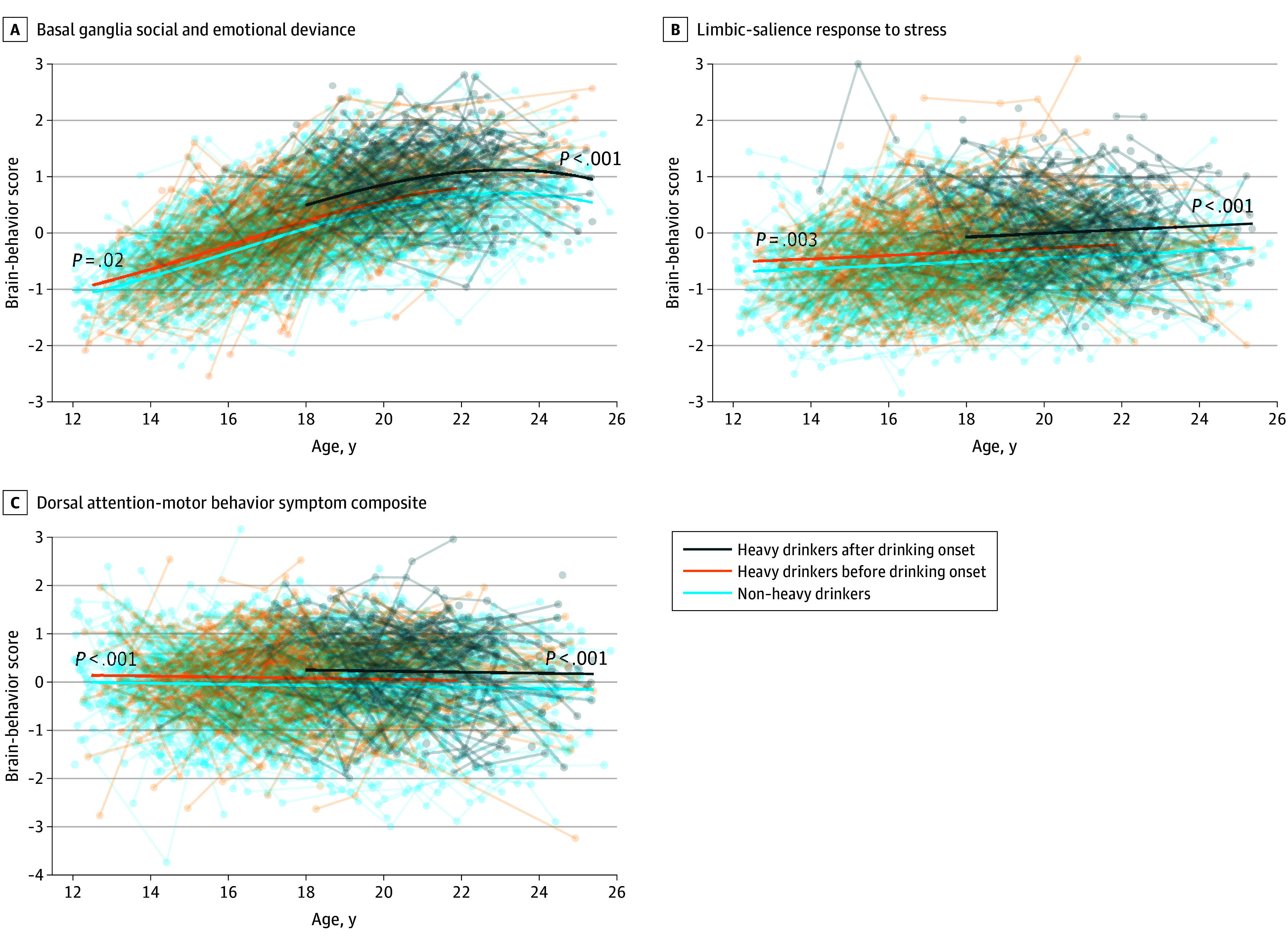
Developmental Trajectories of Brain-Behavior Scores From 3 Socioemotional Components The highest order of the polynomial of age was determined by stepwise regression. *P* values correspond to significant developmental deviation with respect to the non–heavy drinkers.

Compared with non–heavy drinkers, the first brain-behavior score (associated with basal ganglia and anxious and depressive symptoms) showed a significant elevation among heavy drinkers prior to drinking onset (score increase, 0.13; 95% CI, 0.02-0.22; *P* = .02) (Figure 2A). The second brain-behavior score (associated with the limbic-salience network and Response to Stress Questionnaire subscales) also showed a significant elevation (score increase, 0.18; 95% CI, 0.08-0.28; *P* = .003) (Figure 2B). After heavy drinking onset, all 3 brain-behavior scores showed a significant association with heavy drinking (Figure 2A-C). The largest elevation was observed in the second brain-behavior score (score increase, 0.44; 95% CI, 0.33-0.54; *P* < .001). Critically, in all 3 components, the effect size of the group difference was significantly larger after drinking onset than before onset, with the highest increase observed in the first component (*z* = 3.51; *P* < .001). The association between brain-behavior scores and heavy drinking in the 3 components endured after testing differences in trajectories of functional canonical variables alone or neuropsychological canonical variables alone (eTable 4 in Supplement 1).

The significant associations persisted based on different ways of grouping participants into drinkers vs nondrinkers (eTable 5 in Supplement 1); were not biased by the greater percentage of male and White participants in the heavy drinking group (eFigure 6 in Supplement 1); and endured adjustment for data collection site, race, and socioeconomic status (eTable 6 in Supplement 1). Exploratory analyses revealed that brain-behavior scores did not show a dose response after drinking onset, and there was no significant alcohol-site interaction both before and after drinking onset. However, a significant alcohol-sex interaction emerged, with female heavy drinkers showing a significantly larger elevation in the first brain-behavior score compared with male heavy drinkers (*P* = .014, Bonferroni corrected) (eFigure 7 in Supplement 1) prior to drinking onset. This interaction was no longer significant after drinking onset.

Finally, we analyzed which individual neuropsychological measures of the socioemotional system drove the group differences in brain-behavior scores. Testing the association between heavy drinking and measures with significant loadings (Figure 1; eFigure 8 in Supplement 1) revealed that heavy drinking was associated with higher peer group deviance, more changes in social behavior, more rule-breaking behaviors, more intrusive behaviors, and lower withdrawn depression symptom scores (eFigure 9 in Supplement 1). Other than intrusive behavior, the scores showed significant associations both before and after drinking onset, with the effect sizes enlarged following drinking onset (*t*_2516_ >4.1; *P* < .001).

### Executive Control System

**Figure 3.**
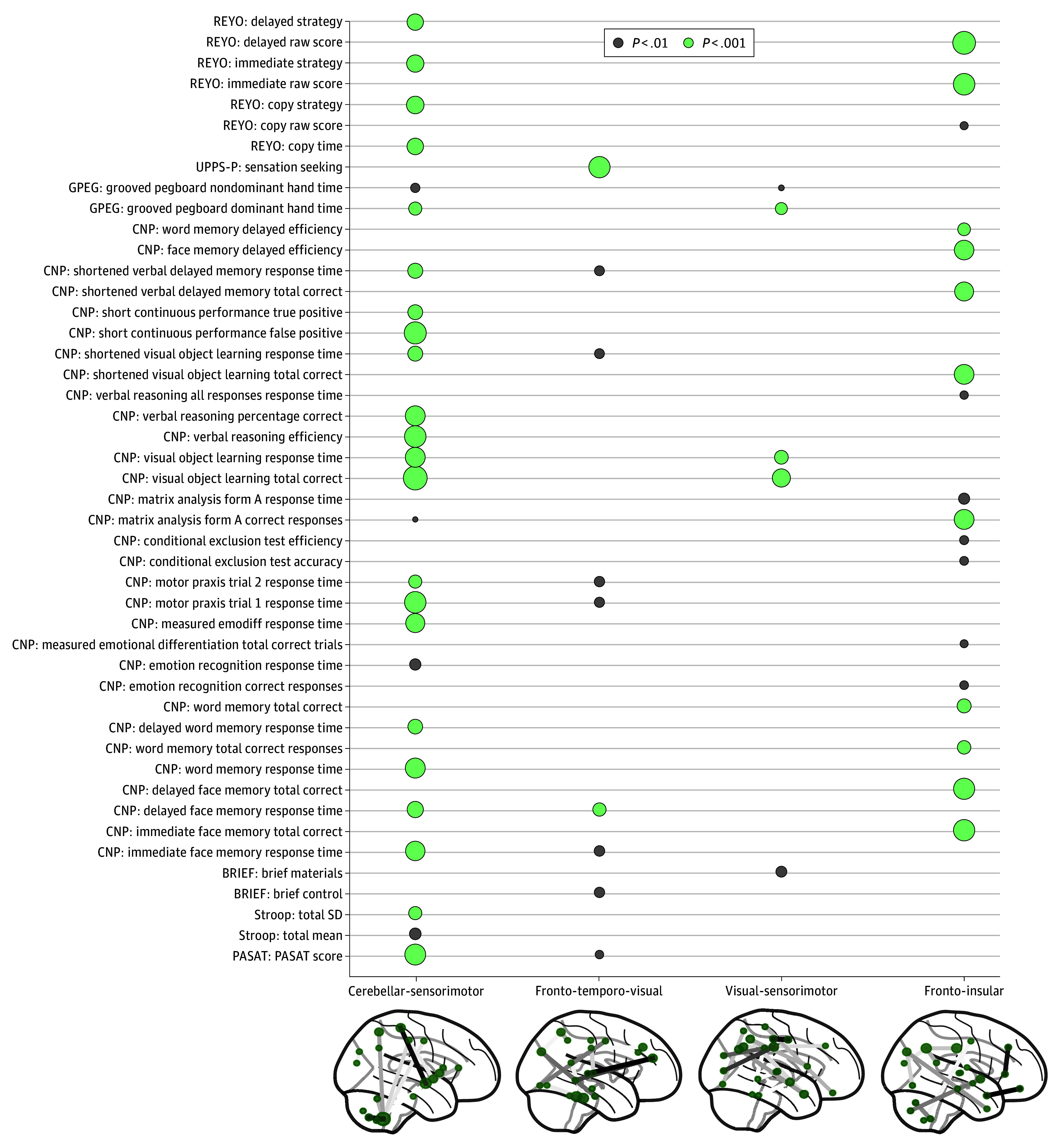
Significant Brain-Behavior Components in the Executive Control Domain and Top 1% Functional Connectivity With Highest Canonical Loadings Each column displays the corresponding neuropsychological measures with significant loadings. Measures with nonsignificant loadings for all 4 components were omitted. BRIEF indicates Behavior Rating Inventory of Executive Function; CNP, Penn Computerized Neurobehavioral Battery; GPEG, grooved pegboard; PASAT, Paced Auditory Serial Addition Test; REYO, Rey-Osterrieth Complex Figure Test; UPPS-P, Urgency-Premeditation-Perseverance-Sensation Seeking-Positive Urgency Impulsive Behavior Scale.

The CCA specific to the executive control system yielded 4 significant components (*P* < .001 by permutation test) (eFigures 4 and 5 in Supplement 1). To ensure that this result was not biased by the larger number of executive control measures (82 vs 44 in the socioemotional system), we reduced the 82 executive control measures to 44 measures by principal component analysis or random sampling and repeated the CCA, which confirmed that all 4 significant components were preserved (eFigure 10 in Supplement 1). Based on the top functional connectivities with the highest canonical loadings (Figure 3; eTable 3 in Supplement 1), the first and the third components captured the functional integration between the sensorimotor network and the cerebellum and visual networks, whereas the second and fourth components captured widespread functional connectivity in the frontal and temporal regions. On examination of significant neuropsychological measures driving the brain-behavior correlation (Figure 3; eFigure 8 in Supplement 1), the first component summarized a wide variety of test scores in the computerized test battery,^31^ the second component was driven by sensation-seeking behavior,^34^ the third component was driven by visual object learning performance, and the fourth component was driven by Rey-Osterrieth complex figure^43^ and face memory test performance.

**Figure 4.**
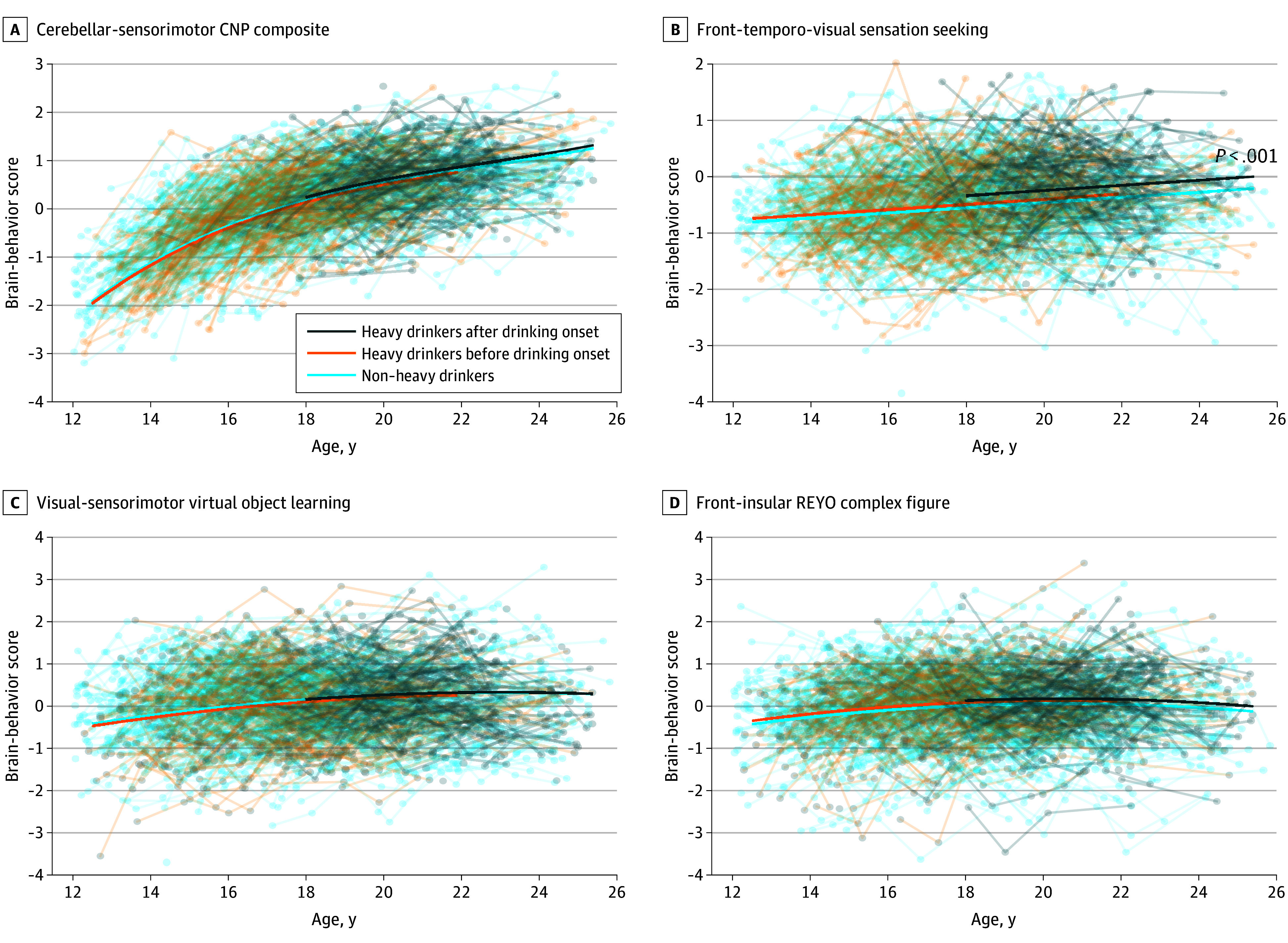
Developmental Trajectories of Brain-Behavior Scores From 4 Executive Control Components The highest order of the polynomial of age was determined by stepwise regression. *P* values correspond to significant developmental deviation with respect to the non–heavy drinkers. CNP indicates Penn Computerized Neurobehavioral Battery; REYO, Rey-Osterrieth Complex Figure Test.

Testing the group difference in the trajectories of brain-behavior scores revealed that compared with non–heavy drinkers, none of the 4 identified components showed significant developmental deviation among the drinkers before they initiated heavy drinking (Figure 4A-D). After drinking onset, however, a significant drinking association emerged for the second brain-behavior score of the executive control system (linked to the fronto-temporo-visual network and sensation-seeking behavior) (score increase, 0.21; 95% CI, 0.11-0.32; *P* < .001) (Figure 4B), in which heavy drinkers showed elevated scores during their heavy drinking visits. This group difference did not significantly interact with sex or site but showed dose responses: After adjusting for age, brain-behavior scores of the heavy drinkers significantly correlated with the average number of drinks (*t*_472_ = 2.7; *P* = .048) and number of binges in the past month (*t*_344_ = 2.8; *P* = .04) (eFigure 11 in Supplement 1).

Finally, applying the same mixed-effects model to regress each of the significant neuropsychological measures of that component revealed that heavy drinkers had significantly higher sensation-seeking scores than non–heavy drinkers (*P* < .001) (eFigure 9F in Supplement 1) both before and after drinking onset and poorer emotion control (score increase, 0.18; 95% CI, 0.04-0.32; *P* for trend = .01) (eFigure 9G in Supplement 1) only after drinking onset.

## Discussion

In this cohort study based on 9-year longitudinal neuroimaging, neuropsychological, and behavioral data of the NCANDA cohort, our data-driven approach yielded neurobiological evidence supporting the association of dual-system asynchronous development with adolescent drinking problems. Heightened reactivity in the socioemotional system during adolescence was found to hijack the regulatory ability of the executive control system, heightening the risk of initiating heavy alcohol consumption. Heavy drinking, in turn, was associated with deficits in the executive control system and exacerbated alteration in the socioemotional system during late adolescence and young adulthood. This pattern may represent a precursor of developing addiction, another cyclical process (ie, the Koob model)^44,45^ driven by neurobiological changes in similar functional systems responsible for reward processing, emotion regulation, and executive control.

The maturational imbalance model is a testable hypothesis that has arisen from developmental cognitive science.^1,2,5^ Several variants of the model exist that diverge in their specific formulations on the shape of developmental trajectory and timing of the maturational peak of brain systems.^46,47,48^ To characterize trajectories, research often has relied on only a few neuropsychological measures, which might not capture the full complexity of a brain-behavior system. Our data-driven analysis enriched the characterization by coupling whole-brain functional connectivity with a comprehensive array of neuropsychological measures. Results showed that the socioemotional component (Figure 2A) was characterized by an inverted *U* shape that plateaued after age 20 years and declined thereafter, whereas the executive control component (Figure 4A) continued to develop into young adulthood. Such distinct developmental patterns between the 2 systems raise the possibility of maturational imbalance.

A major debate poses whether it is possible to measure maturational imbalance objectively.^49^ Indeed, the dual systems were segregated in their functional networks and primary roles,^49^ and neuropsychological measures associated with the 2 systems were often not commensurate. Our analysis detected imbalance by comparing the developmental trajectory of each system with its respective norm. As shown herein, the onset of heavy drinking was associated with elevated brain-behavior strength only in the socioemotional system. Such higher socioemotional reactivity outpaced the regulatory capacity of the executive control system, which followed a typical, slower developmental trajectory without significant deviation.

Regarding the predrinking deviation in the socioemotional system, widespread associations with functional networks and neuropsychological measures were observed in 3 neural systems (Figure 1). In particular, the dorsal attention network is crucial for voluntary attention control,^4,50^ supporting emotional self-regulation, social attention, and response and resilience to stressors.^51,52,53,54,55^ On the other hand, the basal ganglia and limbic systems are critical dopamine pathways involving the nucleus accumbens^56,57^ and play a role in processing rewards, emotions, and social cues.^58,59,60,61^ These functional associations underpinned impaired neuropsychological behaviors in social functioning and mental health problems^62^ (Figure 2A-C; eFigure 9A-E in Supplement 1), increasing the risk of heavy drinking onset, especially in female participants. This finding was in line with a previous analysis of NCANDA participants showing that 27 mental health measures during high school better predicted heavy drinking onset during college among young women compared with young men.^19^

In contrast to the socioemotional system, the association between heavy drinking and brain-behavior scores in the executive control system was primarily observed after drinking onset (Figure 4B). The associated component involved a constellation of neuropsychological measures led by sensation seeking and functional connectivity in regions commonly identified in default mode and sensorimotor networks. Notably, these findings replicated and extended a prior analysis of the first 3 years of longitudinal NCANDA data,^17^ which also showed that heavy drinkers exhibited greater within-network connectivity in the sensorimotor network and 2 other motor networks with more alcohol consumption, a dose response that persisted in the current analysis. Concurrently, the impaired executive control system was less able to regulate the exacerbated stress and negative emotional states.

### Limitations

This study had some limitations. First, our categorization of neuropsychological measures into 2 systems was subjective and required fine-tuning. Many measures cut across both systems; for example, the emotion recognition test in WebCNB,^31,63^ delay discounting, and impulsivity are indicators of both cognitive control and socioemotion.^49^ Additionally, although we showed that principal component analysis and bilateral averaging of functional connectivity could improve canonical correlation between brain and behavior measures, this practice might obscure findings on unilateral functional networks associated with lateralized executive tasks. Finally, our analysis did not consider the influence of genetic variation and fine-grained environmental factors, such as urbanicity and pollution, on maturational imbalance.

## Conclusions

In this cohort study of NCANDA participants, we used a data-driven approach to analyze 9-year longitudinal neuroimaging, neuropsychological, and behavioral data to find direct biological evidence to support the role of dual-system asynchrony in adolescent drinking behaviors. The findings suggest that heightened socioemotional reactivity occurring during adolescence may overwhelm executive control, increasing the risk of heavy alcohol use, which, in turn, may pose risks for developing alcohol-induced damage of both systems. This cycle could enable and reinforce persistent alcohol use, thereby increasing vulnerability to developing alcohol use disorder.

## Notes

Supplement 1.
eMethods.
**eFigure 1.** Schematic Overview of Analysis Workflow **eFigure 2.** Distribution of Age of All Participant Visits **eTable 1.** Neuropsychological Measures of Socioemotional System **eTable 2.** Neuropsychological Measures of the Executive Control System **eFigure 3.** Two CCA Models Used to Find Correlated Patterns Between Resting-State Functional Connectivity and Neuropsychological Measures **eFigure 4.** Distribution of Regression Slope of Linear Mixed-Effects Models **eFigure 5.** Distribution of Canonical Correlation Values Across Processing Methods of Resting-State Functional Data **eTable 3.** Top 5 Functional Connectivities (Between Pairs of Regions) With Highest Canonical Loadings for Each Significant Canonical Component in eFigure 3. **eTable 4.** *P* Values of the Group Differences Between Heavy Drinkers and Non–heavy Drinkers Based on Different Regression Targets **eTable 5.** The Same LME Models in eTable 4 Applied to Different Groupings of Participants Into Drinkers (Cahalan Score > 0) and Nondrinkers (Cahalan Score = 0) **eTable 6.** Results of LME With Additional Covariates of Race, Site, and Socioeconomic Status **eFigure 6.** Distribution of *P* Values (Uncorrected) Associated With the Alcohol Effects When Running Mixed-Effects Models on Randomly Sampled Cohorts **eFigure 7.** Significant Sex-Alcohol Interaction in the First Socioemotional Component **eFigure 8.** Null Distribution of Canonical Loadings for Individual Neuropsychological Measures of the Socioemotional and Executive Control Systems **eFigure 9.** Distribution of Top Neuropsychological Measures Defining the Canonical Components That Showed Significant Alcohol Effects **eFigure 10.** Distributions of Canonical Correlations **eFigure 11. **Brain-Behavior Score of the Second Component of the Executive Control System
eReferences.

Supplement 2.
Data Sharing Statement

## References

[1] Casey B, Jones RM, Somerville LH. Braking and accelerating of the adolescent brain. J Res Adolesc. 2011;21(1):21-33. doi:10.1111/j.1532-7795.2010.00712.x 21475613 PMC3070306

[2] Steinberg L. A dual systems model of adolescent risk-taking. Dev Psychobiol. 2010;52(3):216-224. doi:10.1002/dev.20445 20213754

[3] Pfeifer JH, Peake SJ. Self-development: integrating cognitive, socioemotional, and neuroimaging perspectives. Dev Cogn Neurosci. 2012;2(1):55-69. doi:10.1016/j.dcn.2011.07.012 22682728 PMC6987679

[4] Hart G, Leung BK, Balleine BW. Dorsal and ventral streams: the distinct role of striatal subregions in the acquisition and performance of goal-directed actions. Neurobiol Learn Mem. 2014;108:104-118. doi:10.1016/j.nlm.2013.11.003 24231424 PMC4661143

[5] Shulman EP, Smith AR, Silva K, . The dual systems model: review, reappraisal, and reaffirmation. Dev Cogn Neurosci. 2016;17:103-117. doi:10.1016/j.dcn.2015.12.010 26774291 PMC6990093

[6] Miller EK, Cohen JD. An integrative theory of prefrontal cortex function. Annu Rev Neurosci. 2001;24:167-202. doi:10.1146/annurev.neuro.24.1.167 11283309

[7] Nowrangi MA, Lyketsos C, Rao V, Munro CA. Systematic review of neuroimaging correlates of executive functioning: converging evidence from different clinical populations. J Neuropsychiatry Clin Neurosci. 2014;26(2):114-125. doi:10.1176/appi.neuropsych.12070176 24763759 PMC5171230

[8] Tervo-Clemmens B, Calabro FJ, Parr AC, Fedor J, Foran W, Luna B. A canonical trajectory of executive function maturation from adolescence to adulthood. Nat Commun. 2023;14(1):6922. doi:10.1038/s41467-023-42540-8 37903830 PMC10616171

[9] Satterthwaite TD, Wolf DH, Erus G, . Functional maturation of the executive system during adolescence. J Neurosci. 2013;33(41):16249-16261. doi:10.1523/JNEUROSCI.2345-13.2013 24107956 PMC3792462

[10] Pfefferbaum A, Kwon D, Brumback T, . Altered brain developmental trajectories in adolescents after initiating drinking. Am J Psychiatry. 2018;175(4):370-380. doi:10.1176/appi.ajp.2017.17040469 29084454 PMC6504929

[11] El Marroun H, Klapwijk ET, Koevoets M, . Alcohol use and brain morphology in adolescence: a longitudinal study in three different cohorts. Eur J Neurosci. 2021;54(6):6012-6026. doi:10.1111/ejn.15411 34390509 PMC9291789

[12] Squeglia LM, Tapert SF, Sullivan EV, . Brain development in heavy-drinking adolescents. Am J Psychiatry. 2015;172(6):531-542. doi:10.1176/appi.ajp.2015.14101249 25982660 PMC4451385

[13] Zhao Q, Sullivan EV, Honnorat N, . Association of heavy drinking with deviant fiber tract development in frontal brain systems in adolescents. JAMA Psychiatry. 2021;78(4):407-415. doi:10.1001/jamapsychiatry.2020.4064 33377940 PMC7774050

[14] McQueeny T, Schweinsburg BC, Schweinsburg AD, . Altered white matter integrity in adolescent binge drinkers. Alcohol Clin Exp Res. 2009;33(7):1278-1285. doi:10.1111/j.1530-0277.2009.00953.x 19389185 PMC2825379

[15] Müller-Oehring EM, Kwon D, Nagel BJ, . Influences of age, sex, and moderate alcohol drinking on the intrinsic functional architecture of adolescent brains. Cereb Cortex. 2018;28(3):1049-1063. doi:10.1093/cercor/bhx014 28168274 PMC6059181

[16] Fang X, Deza-Araujo YI, Petzold J, . Effects of moderate alcohol levels on default mode network connectivity in heavy drinkers. Alcohol Clin Exp Res. 2021;45(5):1039-1050. doi:10.1111/acer.14602 33742481

[17] Zhao Q, Sullivan EV, Müller-Oehring EM, . Adolescent alcohol use disrupts functional neurodevelopment in sensation seeking girls. Addict Biol. 2021;26(2):e12914. doi:10.1111/adb.12914 32428984 PMC7883631

[18] Yip SW, Lichenstein SD, Liang Q, . Brain networks and adolescent alcohol use. JAMA Psychiatry. 2023;80(11):1131-1141. doi:10.1001/jamapsychiatry.2023.2949 37647053 PMC10469292

[19] Zhao Q, Paschali M, Dehoney J, . Identifying high school risk factors that forecast heavy drinking onset in understudied young adults. Dev Cogn Neurosci. 2024;68:101413. doi:10.1016/j.dcn.2024.101413 38943839 PMC11261404

[20] Lees B, Meredith LR, Kirkland AE, Bryant BE, Squeglia LM. Effect of alcohol use on the adolescent brain and behavior. Pharmacol Biochem Behav. 2020;192:172906. doi:10.1016/j.pbb.2020.172906 32179028 PMC7183385

[21] Meda SA, Dager AD, Hawkins KA, . Heavy drinking in college students is associated with accelerated gray matter volumetric decline over a 2 year period. Front Behav Neurosci. 2017;11:176. doi:10.3389/fnbeh.2017.00176 29033801 PMC5627037

[22] Brown SA, Brumback T, Tomlinson K, . The National Consortium on Alcohol and NeuroDevelopment in Adolescence (NCANDA): a multisite study of adolescent development and substance use. J Stud Alcohol Drugs. 2015;76(6):895-908. doi:10.15288/jsad.2015.76.895 26562597 PMC4712659

[23] Cahalan D, Cisin IH, Crossley HM. *American Drinking Practices: A National Study of Drinking Behavior and Attitudes*. Rutgers Monograph Series 6. Center of Alcohol Studies at Yale and Rutgers; 1969.

[24] Sullivan EV, Lane B, Kwon D, . Structural brain anomalies in healthy adolescents in the NCANDA cohort: relation to neuropsychological test performance, sex, and ethnicity. Brain Imaging Behav. 2017;11(5):1302-1315. doi:10.1007/s11682-016-9634-2 27722828 PMC5656437

[25] Rohlfing T, Zahr NM, Sullivan EV, Pfefferbaum A. The SRI24 multichannel atlas of normal adult human brain structure. Hum Brain Mapp. 2010;31(5):798-819. doi:10.1002/hbm.20906 20017133 PMC2915788

[26] Toro R, Fox PT, Paus T. Functional coactivation map of the human brain. Cereb Cortex. 2008;18(11):2553-2559. doi:10.1093/cercor/bhn014 18296434 PMC2567424

[27] Sullivan EV, Brumback T, Tapert SF, . Cognitive, emotion control, and motor performance of adolescents in the NCANDA study: contributions from alcohol consumption, age, sex, ethnicity, and family history of addiction. Neuropsychology. 2016;30(4):449–473. doi:10.1037/neu0000259 26752122 PMC4840074

[28] Pfefferbaum A, Rohlfing T, Pohl KM, . Adolescent development of cortical and white matter structure in the NCANDA sample: role of sex, ethnicity, puberty, and alcohol drinking. Cereb Cortex. 2016;26(10):4101-4121. doi:10.1093/cercor/bhv205 26408800 PMC5027999

[29] Wang HT, Smallwood J, Mourao-Miranda J, . Finding the needle in a high-dimensional haystack: canonical correlation analysis for neuroscientists. Neuroimage. 2020;216:116745. doi:10.1016/j.neuroimage.2020.116745 32278095

[30] Zheng J, Rakovski C. On the application of principal component analysis to classification problems. Data Sci J. 2021;20:1-6. doi:10.5334/dsj-2021-026

[31] Gur RC, Richard J, Hughett P, . A cognitive neuroscience-based computerized battery for efficient measurement of individual differences: standardization and initial construct validation. J Neurosci Methods. 2010;187(2):254-262. doi:10.1016/j.jneumeth.2009.11.017 19945485 PMC2832711

[32] Odum AL. Delay discounting: trait variable? Behav Processes. 2011;87(1):1-9. doi:10.1016/j.beproc.2011.02.007 21385637 PMC3266171

[33] Schmidt SL, Oliveira RM, Rocha FR, Abreu-Villaça Y. Influences of handedness and gender on the grooved pegboard test. Brain Cogn. 2000;44(3):445-454. doi:10.1006/brcg.1999.1204 11104536

[34] Cyders MA, Littlefield AK, Coffey S, Karyadi KA. Examination of a short English version of the UPPS-P Impulsive Behavior Scale. Addict Behav. 2014;39(9):1372-1376. doi:10.1016/j.addbeh.2014.02.013 24636739 PMC4055534

[35] Gioia GA, Isquith PK, Retzlaff PD, Espy KA. Confirmatory factor analysis of the Behavior Rating Inventory of Executive Function (BRIEF) in a clinical sample. Child Neuropsychol. 2002;8(4):249-257. doi:10.1076/chin.8.4.249.13513 12759822

[36] Brown SA, Christiansen BA, Goldman MS. The Alcohol Expectancy Questionnaire: an instrument for the assessment of adolescent and adult alcohol expectancies. J Stud Alcohol. 1987;48(5):483-491. doi:10.15288/jsa.1987.48.483 3669677

[37] Sturz B, Green ML, Locker L Jr, Boyer TW. Stroop interference in a delayed match-to-sample task: evidence for semantic competition. Front Psychol. 2013;4:842. doi:10.3389/fpsyg.2013.00842 24298264 PMC3828616

[38] knnimpute. MATLAB Help Center. Accessed July 3, 2025. https://www.mathworks.com/help/bioinfo/ref/knnimpute.html

[39] Smith SM, Nichols TE, Vidaurre D, . A positive-negative mode of population covariation links brain connectivity, demographics and behavior. Nat Neurosci. 2015;18(11):1565-1567. doi:10.1038/nn.4125 26414616 PMC4625579

[40] Efroymson MA. Multiple Regression Analysis. Mathematical Methods for Digital Computers. Wiley; 1960.

[41] Connor-Smith JK, Compas BE, Wadsworth ME, Thomsen AH, Saltzman H. Responses to stress in adolescence: measurement of coping and involuntary stress responses. J Consult Clin Psychol. 2000;68(6):976-992. doi:10.1037/0022-006X.68.6.976 11142550

[42] Lacalle Sisteré M, Domènech Massons JM, Granero Pérez R, Ezpeleta Ascaso L. Validity of the DSM-oriented scales of the Child Behavior Checklist and Youth Self-Report. Psicothema. 2014;26(3):364-371. doi:10.7334/psicothema2013.342 25069556

[43] Shin MS, Park SY, Park SR, Seol SH, Kwon JS. Clinical and empirical applications of the Rey-Osterrieth Complex Figure Test. Nat Protoc. 2006;1(2):892-899. doi:10.1038/nprot.2006.115 17406322

[44] Kwako LE, Schwandt ML, Ramchandani VA, . Neurofunctional domains derived from deep behavioral phenotyping in alcohol use disorder. Am J Psychiatry. 2019;176(9):744-753. doi:10.1176/appi.ajp.2018.18030357 30606047 PMC6609498

[45] Voon V, Grodin E, Mandali A, . Addictions NeuroImaging Assessment (ANIA): towards an integrative framework for alcohol use disorder. Neurosci Biobehav Rev. 2020;113:492-506. doi:10.1016/j.neubiorev.2020.04.004 32298710

[46] Casey BJ, Jones RM, Hare TA. The adolescent brain. Ann N Y Acad Sci. 2008;1124:111-126. doi:10.1196/annals.1440.010 18400927 PMC2475802

[47] Steinberg L. A social neuroscience perspective on adolescent risk-taking. Dev Rev. 2008;28(1):78-106. doi:10.1016/j.dr.2007.08.002 18509515 PMC2396566

[48] Luna B, Wright C. Adolescent Brain Development: Implications to the Juvenile Criminal Justice System. American Psychological Association; 2016.

[49] Meisel SN, Fosco WD, Hawk LW, Colder CR. Mind the gap: a review and recommendations for statistically evaluating dual systems models of adolescent risk behavior. Dev Cogn Neurosci. 2019;39:100681. doi:10.1016/j.dcn.2019.100681 31404858 PMC6969358

[50] Choi EJ, Vandewouw MM, de Villa K, Inoue T, Taylor MJ. The development of functional connectivity within the dorsal striatum from early childhood to adulthood. Dev Cogn Neurosci. 2023;61:101258. doi:10.1016/j.dcn.2023.101258 37247471 PMC10236186

[51] Kaiser RH, Andrews-Hanna JR, Wager TD, Pizzagalli DA. Large-scale network dysfunction in major depressive disorder: a meta-analysis of resting-state functional connectivity. JAMA Psychiatry. 2015;72(6):603-611. doi:10.1001/jamapsychiatry.2015.0071 25785575 PMC4456260

[52] Viviani R. Emotion regulation, attention to emotion, and the ventral attentional network. Front Hum Neurosci. 2013;7:746. doi:10.3389/fnhum.2013.00746 24223546 PMC3819767

[53] Lee D, Lee J, Namkoong K, Jung YC. Altered functional connectivity of the dorsal attention network among problematic social network users. Addict Behav. 2021;116:106823. doi:10.1016/j.addbeh.2021.106823 33460991

[54] Broeders TAA, Schoonheim MM, Vink M, . Dorsal attention network centrality increases during recovery from acute stress exposure. Neuroimage Clin. 2021;31:102721. doi:10.1016/j.nicl.2021.102721 34134017 PMC8214139

[55] Teng J, Massar SAA, Lim J. Inter-relationships between changes in stress, mindfulness, and dynamic functional connectivity in response to a social stressor. Sci Rep. 2022;12(1):2396. doi:10.1038/s41598-022-06342-0 35165343 PMC8844001

[56] Ikemoto S, Yang C, Tan A. Basal ganglia circuit loops, dopamine and motivation: a review and enquiry. Behav Brain Res. 2015;290:17-31. doi:10.1016/j.bbr.2015.04.018 25907747 PMC4447603

[57] Goto Y, Grace AA. Dopaminergic modulation of limbic and cortical drive of nucleus accumbens in goal-directed behavior. Nat Neurosci. 2005;8(6):805-812. doi:10.1038/nn1471 15908948

[58] Pierce JE, Péron J. The basal ganglia and the cerebellum in human emotion. Soc Cogn Affect Neurosci. 2020;15(5):599-613. doi:10.1093/scan/nsaa076 32507876 PMC7328022

[59] Šimić G, Tkalčić M, Vukić V, . Understanding emotions: origins and roles of the amygdala. Biomolecules. 2021;11(6):823. doi:10.3390/biom11060823 34072960 PMC8228195

[60] Vega-Zelaya L, Pastor J. The network systems underlying emotions: the rational foundation of deep brain stimulation psychosurgery. Brain Sci. 2023;13(6):943. doi:10.3390/brainsci13060943 37371421 PMC10296681

[61] Ceravolo L, Frühholz S, Pierce J, Grandjean D, Péron J. Basal ganglia and cerebellum contributions to vocal emotion processing as revealed by high-resolution fMRI. Sci Rep. 2021;11(1):10645. doi:10.1038/s41598-021-90222-6 34017050 PMC8138027

[62] Moore A, Lewis B, Elton A, Squeglia LM, Nixon SJ. An investigation of multimodal predictors of adolescent alcohol initiation. Drug Alcohol Depend. 2024;265:112491. doi:10.1016/j.drugalcdep.2024.112491 39522301 PMC12444771

[63] Gur RE, Kohler CG, Ragland JD, . Flat affect in schizophrenia: relation to emotion processing and neurocognitive measures. Schizophr Bull. 2006;32(2):279-287. doi:10.1093/schbul/sbj041 16452608 PMC2632232

