# Error in Funding/Support

**DOI:** 10.1001/jamanetworkopen.2025.39577

**Published:** 2025-10-07

In the Original Investigation titled “Socioemotional and Executive Control Mismatch in Adolescence and Risks for Initiating Drinking,”^1^ published September 12, 2025, there was an error in the presentation of grants in the Funding/Support section of the Article Information. The statement, “Data collection and management were supported by grant U24AA021697 from the National Institute of Alcohol Abuse and Alcoholism,” was replaced with, “Collection and distribution of the NCANDA data were supported by grants AA021681, AA021690, AA021691, AA021692, AA021695, AA021696, and AA021697 from the National Institutes of Health.” This article has been corrected.^1^

## References

[1] Zhao Q, Milecki L, Kuceyeski A, . Socioemotional and executive control mismatch in adolescence and risks for initiating drinking. JAMA Netw Open. 2025;8(10):e2531378. doi:10.1001/jamanetworkopen.2025.31378 40938600 PMC12432642

