# Error in Abstract

**DOI:** 10.1001/jamanetworkopen.2026.18787

**Published:** 2026-06-02

In the Original Investigation titled “Socioemotional and Executive Control Mismatch in Adolescence and Risks for Initiating Drinking,”^1^ published September 12, 2025, the Abstract Results were revised to clarify differences in the timing at which developmental deviations emerge between the excutive control and socioemotional systems. This article has been corrected.^1^
