# Supplementary material for: Socioemotional and Executive Control Mismatch in Adolescence and Risks for Initiating Drinking
Source: JAMA Netw Open. 2025 Sep 12;8(9):e2531378. doi: 10.1001/jamanetworkopen.2025.31378 (PMC12432642; doi:10.1001/jamanetworkopen.2025.31378)
Supplement: Supplement 1. — eMethods. eFigure 1. Schematic Overview of Analysis Workflow eFigure 2. Distribution of Age of All Participant Visits eTable 1. Neuropsychological Measures of Socioemotional System eTable 2. Neuropsychological Measures of the Executive Control System eFigure 3. Two CCA Models Used to Find Correlated Patterns Between Resting-State Functional Connectivity and Neuropsychological Measures eFigure 4. Distribution of Regression Slope of Linear Mixed-Effects Models eFigure 5. Distribution of Canonical Correlation Values Across Processing Methods of Resting-State Functional Data eTable 3. Top 5 Functional Connectivities (Between Pairs of Regions) With Highest Canonical Loadings for Each Significant Canonical Component in eFigure 3. eTable 4. P Values of the Group Differences Between Heavy Drinkers and Non–heavy Drinkers Based on Different Regression Targets eTable 5. The Same LME Models in eTable 4 Applied to Different Groupings of Participants Into Drinkers (Cahalan Score > 0) and Nondrinkers (Cahalan Score = 0) eTable 6. Results of LME With Additional Covariates of Race, Site, and Socioeconomic Status eFigure 6. Distribution of P Values (Uncorrected) Associated With the Alcohol Effects When Running Mixed-Effects Models on Randomly Sampled Cohorts eFigure 7. Significant Sex-Alcohol Interaction in the First Socioemotional Component eFigure 8. Null Distribution of Canonical Loadings for Individual Neuropsychological Measures of the Socioemotional and Executive Control Systems eFigure 9. Distribution of Top Neuropsychological Measures Defining the Canonical Components That Showed Significant Alcohol Effects eFigure 10. Distributions of Canonical Correlations eFigure 11. Brain-Behavior Score of the Second Component of the Executive Control System eReferences. [file jamanetwopen-e2531378-s001.pdf]

Supplemental Online Content

Zhao Q, Milecki L, Kuceyeski A, et al. Socioemotional and executive control mismatch in adolescence and risks for initiating drinking. *JAMA Netw Open*. 2025:8(9):e2531378. doi:10.1001/jamanetworkopen.2025.31378

eMethods.

eFigure 1. Schematic Overview of Analysis Workflow

eFigure 2. Distribution of Age of All Participant Visits

eTable 1. Neuropsychological Measures of Socioemotional System

eTable 2. Neuropsychological Measures of the Executive Control System

eFigure 3. Two CCA Models Used to Find Correlated Patterns Between Resting-State Functional Connectivity and Neuropsychological Measures

eFigure 4. Distribution of Regression Slope of Linear Mixed-Effects Models

eFigure 5. Distribution of Canonical Correlation Values Across Processing Methods of Resting-State Functional Data

eTable 3. Top 5 Functional Connectivities (Between Pairs of Regions) With Highest Canonical Loadings for Each Significant Canonical Component in eFigure 3.

eTable 4. *P* Values of the Group Differences Between Heavy Drinkers and Nonheavy Drinkers Based on Different Regression Targets

eTable 5. The Same LME Models in eTable 4 Applied to Different Groupings of Participants Into Drinkers (Cahalan Score > 0) and Nondrinkers (Cahalan Score = 0)

eTable 6. Results of LME With Additional Covariates of Race, Site, and Socioeconomic Status

eFigure 6. Distribution of *P* Values (Uncorrected) Associated With the Alcohol Effects When Running Mixed-Effects Models on Randomly Sampled Cohorts

eFigure 7. Significant Sex-Alcohol Interaction in the First Socioemotional Component

eFigure 8. Null Distribution of Canonical Loadings for Individual Neuropsychological Measures of the Socioemotional and Executive Control Systems

eFigure 9. Distribution of Top Neuropsychological Measures Defining the Canonical Components That Showed Significant Alcohol Effects

eFigure 10. Distributions of Canonical Correlations

eFigure 11. Brain-Behavior Score of the Second Component of the Executive Control System

eReferences.

This supplemental material has been provided by the authors to give readers additional information about their work.

eMethods

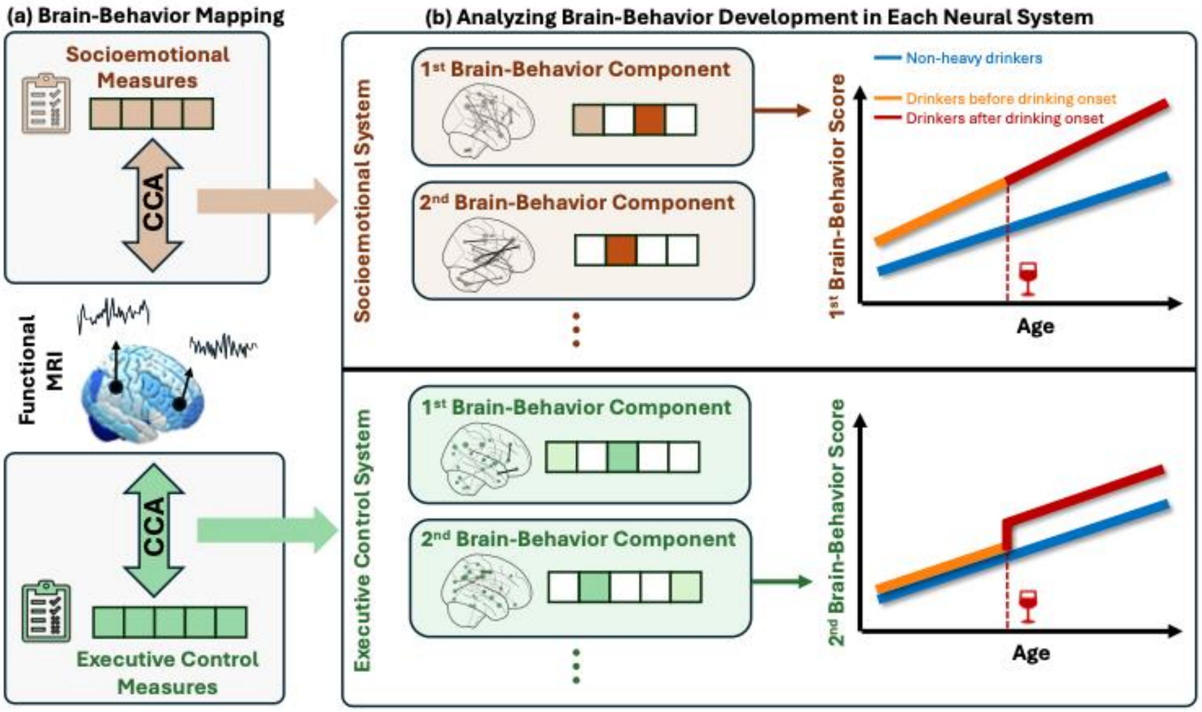

eFigure 1. **Schematic Overview of Analysis Workflow:** (a) two CCA models extracted correlated patterns between resting-state functional connectivity and neuropsychological measures from the dual systems; (b) Each CCA resulted in a set of significant brain-behavior components encoding the coupling between a set of functional connectivity and a constellation of neuropsychological measures. For each component, a brain-behavior score quantified its strength at a participant visit. Then mixed-effect models compared the trajectories of the brain-behavior scores among non-heavy drinkers to those of heavy drinkers, before and after drinking onset. We hypothesize that only the socioemotional system would show faster development (elevated score) before drinking onset, whereas the executive control system followed the norm. Such imbalance would heighten the risk of heavy drinking onset, which resulted in more intense effects on the socioemotional system and impaired executive control function.

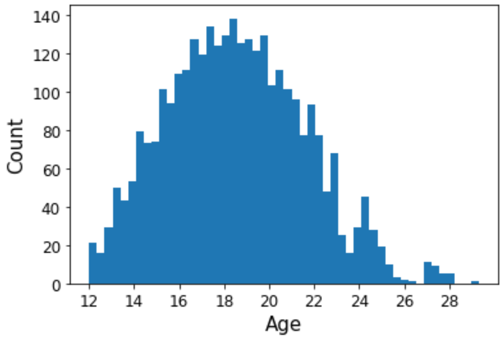

eFigure 2. Distribution of Age of All Participant Visits. The right tail of the distribution (visits above age 26 years) was omitted in the CCA analysis

© 2025 Zhao Q et al. *JAMA Network Open*.

Canonical Correlation Analysis. Canonical Correlation Analysis (CCA) [4] is a statistical technique used to explore the relationships between two sets of variables (e.g., functional connectivity and neuropsychological measures). It quantifies the associations between the two variable sets by finding pairs of linear combinations that are maximally correlated. More formally, we suppose we have two sets of measurements X, Y from n samples, where X is a $p * n$ matrix (p-dimensional brain connectivity features from rs-fMRI data of $n = 3076$ participant visits), and Y is a $q * n$ matrix (q-dimensional neuropsychological measures of n participant visits). CCA forms linear transformations a and b such that $U = aX$ and $V = bY$, where U and V are called the Canonical Variables of the n samples. CCA finds a and b such that the correlation between U and V is maximized: $r = corr(U, V)$. The first canonical variable pair (U_1, V_1) captures the strongest relationship between the two datasets. Subsequent canonical pairs, or components, (U_2, V_2), (U_3, V_3), *etc.* are computed sequentially, subject to being uncorrelated with previous pairs. Each pair represents a new dimension of brain-behavior association. Key concepts include:

1. Canonical Correlation r_i measures the strength of association between the two canonical variables of the ith component.
2. Brain-Behavior Score: For each sample (each participant visit), the functional canonical variable and neuropsychological canonical variable associated with a canonical component records the reactivity strength of that component. We then take the z-score of the functional canonical variable and the z-score of the neuropsychological canonical variable and average the two z-scores as the brain-behavior score defined per subject – visit, i.e., $(z_{score}(U_i) + z_{score}(V_i))/2$ defines the ith brain-behavior score for the $n = 3076$ participant visits
3. Canonical Loading of variable j (the jth functional measure or the jth neuropsychological measure) for component i is defined as the correlation between the original j^{th} variable in X (or Y) and the ith canonical variable, *i.e., $loading(X_{i,j}) = corr(x_j, U_i)$* and $loading(Y_{i,j}) = corr(y_j, V_i)$. A high loading $X_{i,j}$ (or $Y_{i,j}$) indicates that the jth functional (or neuropsychological) measure plays a major role in defining the ith canonical component. Note, functional loadings were initially computed for the 384 PCA scores, which were then projected back to the original space resulting in loadings for 1378 functional connections.

Permutation Tests: A permutation test was separately performed for the socioemotional system and the executive control system. For either system, we performed a subject-level permutation after preprocessing the functional connectivity and neuropsychological data. Then we performed a 10-fold cross validation of the CCA model on the permuted data. We repeated the permutation 20 times, which resulted in 20x10x44 canonical correlation values (# of permutation x # of folds x # of canonical components) for the socioemotional system and 20x10x82 correlation values for the executive system. We observed that the distribution of correlation values was virtually identical across different canonical components, so we built a single null distribution of correlation values for each system (eFigure 3). Similarly, we derived the neuropsychological loadings across all folds and permutations and built a single null distribution of the loading values for each system (eFigure 8).

© 2025 Zhao Q et al. *JAMA Network Open*.

eTable 1. Neuropsychological Measures of the Socioemotional System

Test Battery	Measurement Name
Alcohol Expectancy Questionnaire [5]	Changes in Social Behavior
	Global Positive Change
	Increased Arousal
	Improved Cognitive and Motor Ability
	Relaxation and Tension Reduction
	Alcohol Outcome Expectancies - Total Score
	Sexual Enhancement
ASEBA [6]	Aggressive Behavior
	Anxious/Depressed
	Attention problems
	Social problems
	Rule-breaking behavior
	Somatic complaints
	Thought problems
	Withdrawn/Depressed
Center for Epidemiologic Studies-Depression [7]	CES-D Score
Karolinska Sleepiness Scale [8]	After MRI
	Before MRI
Life Experiences Questionnaire [9]	Common Negative Composite
	Common Chronic Negative Controllable Scale
	Common Chronic Negative Uncontrollable Scale
	Common Discrete Ambiguous Uncontrollable Scale
	Common Discrete Challenging Uncontrollable Composite
	Common Discrete Negative Controllable Scale
	Common Discrete Negative Uncontrollable Scale
	Common Discrete Positive Controllable Scale
	Common Negative Controllable Composite
	Common Negative Uncontrollable Composite
	Common Strictly Negative Composite
	Common Uncontrollable Composite
Peer Group Deviance Score	Peer Group Deviance Score
Response to Stress Questionnaire [10]	RSQ: Acceptance subscale
	RSQ: Cognitive Restructuring subscale
	RSQ: Distraction subscale
	RSQ: Emotion Expression subscale
	RSQ: Emotion Regulation subscale
	RSQ: Positive Thinking subscale
	RSQ: Problem Solving subscale

© 2025 Zhao Q et al. *JAMA Network Open*.

Social Support Questionnaire [11]	SSQ Score
Ten Item Personality Measure [12]	Agreeableness<br>Conscientiousness<br>Emotional Stability<br>Extraversion<br>Openness to Experiences

eTable 2. Neuropsychological Measures of the Executive Control System

Test Battery	Sub-domain (specific to CNB)	Measurement Name
CNB [14]	Complex Cognition	Verbal Reasoning All Responses Median Response Time (ms)<br>Verbal Reasoning Efficiency<br>Matrix Analysis Test Median Response Time for All Responses (ms) for Form A<br>Matrix Analysis Test Correct Responses for Form A<br>Verbal Reasoning Percent Correct
	Episodic Memory	Shortened Verbal Delayed Memory Test Median Response Time for Incorrect Responses (ms)<br>Shortened Verbal Delayed Memory Test Median Response Time for Correct Responses (ms)<br>Shortened Verbal Delayed Memory Test Delayed Memory Total Correct<br>Shortened Visual Object Learning Test Median Response Time for All Responses (ms)<br>Shortened Visual Object Learning Test Total Correct<br>Visual Object Learning Median Response Time for correct trials (ms)<br>Face Memory Median Total Correct Response Time (ms)<br>Visual Object Learning Total Correct Responses<br>Delayed Word Memory Response Time for Total Correct Responses (ms)<br>Delayed Word Memory Total Correct Responses<br>Word Memory Median Response Time for Total Correct Responses (ms)<br>Word Memory Total Correct Responses<br>Median Response Time for CPW Total Correct Responses (ms)<br>Face Memory Total Correct Responses<br>Facial Memory Test-Delayed Efficiency<br>Facial Memory Test-Delayed Median Total Correct Response Time (ms)<br>Facial Memory Test-Delayed Total Correct Responses<br>Visual Object Learning Total Non-Responses
	Executive Function	Short Continuous Performance Test Median Response Time for True Positive Responses (ms)<br>Short Continuous Performance Test True Positives<br>Short Continuous Performance Test Median Response Time for False Positive Responses (ms)<br>Short Continuous Performance Test False Positives

© 2025 Zhao Q et al. *JAMA Network Open*.

		Short Fractal N-Back True Positive Responses<br>Conditional Exclusion Test Efficiency<br>Short Fractal N-Back False Positive Responses<br>Conditional Exclusion Test Median Response Time for Correct Responses (ms)<br>Conditional Exclusion Test Accuracy<br>Short Fractal N-Back Median Response Time for All Correct Responses (ms)
	Sensorimotor Speed	Motor Praxis Trial 2 Median Response Time Correct Responses (ms)<br>Motor Praxis Trial 1 Median Response Time (ms)
	Social Cognition	Measured Emodiff Median Response Time for All Trials<br>Measured Emodiff Total Correct Trials<br>Emotion Recognition Task Correct Responses<br>Emotion Recognition Task Correct Responses Median Response Time (ms)
Delay Discounting [15]		log(k) One Day<br>log(k) One Month<br>log(k) Six Months<br>log(k) One Week<br>log(k) One Day<br>log(k) One Month<br>log(k) Six Months<br>log(k) One Week
Grooved Pegboard [16]		Dominant Hand Time<br>Dominant Hand Drops<br>Non-Dominant Hand Time<br>Non-Dominant Hand Drops
Rey–Osterrieth complex figure [17]		Copy - Final Raw Score<br>Copy - Final Strategy Score<br>Copy - Time<br>Delayed - Final Raw Score<br>Delayed - Final Strategy Score<br>Delayed - Time<br>Elapsed Time Between Immediate and Delayed Trials<br>Immediate - Final Raw Score<br>Immediate - Final Strategy Score<br>Immediate - Time
Paced Auditory Serial Addition Test [18]		Pasat Score
Stroop [19]		Averaged RT over all correct trials in msec

© 2025 Zhao Q et al. *JAMA Network Open*.

	Standard deviation over all correct trials in msec
UPPS-P Impulsive Behavior Scale [20]	Negative Urgency
	Premeditation
	Perseverance
	Positive Urgency
	Sensation Seeking
BRIEF [13]	Behavioral Shift Change Flexibility
	Behavioral Regulation Index (BRI) (raw)
	Cognitive Shift Problem-Solving Flexibility (raw)
	Emotional Control (raw)
	Global Executive Composite (GEC) (raw)
	Inhibitory Control and Impulsivity (raw)
	Materials Orderliness of Environment (raw)
	Working Memory Functionality (raw)
	Metacognition Index (MCI) (raw)
	Monitor Work-checking and Mindfulness (raw)
	Plan/Organize Tasks (raw)
	Behavioral plus Cognitive Flexibility
	Task-Completion Ability to Initiate Strategies

© 2025 Zhao Q et al. *JAMA Network Open*.

Results

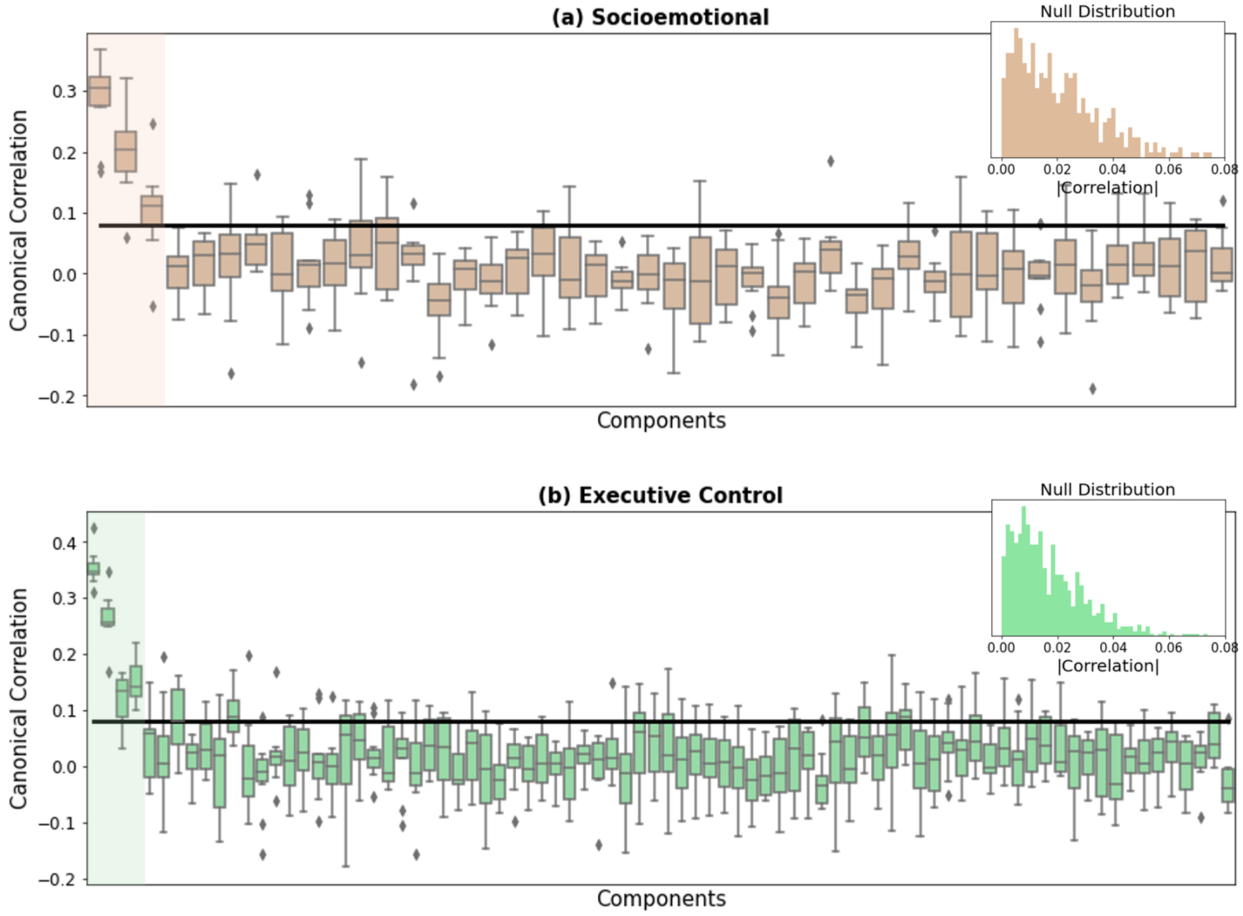

eFigure 3. Canonical Correlation Analysis (CCA) Models Used to Find Correlated Patterns Between Resting-State Functional Connectivity and Neuropsychological Measures. Two CCA models were used to find correlated patterns between resting-state functional connectivity and neuropsychological measures from (a) socioemotional or (b) executive control system. Distributions of canonical correlation (between functional and neuropsychological canonical variables) associated with all canonical components were measured on the 10 testing folds and compared with the null threshold at the p=0.0001 level (black line).

© 2025 Zhao Q et al. *JAMA Network Open*.

Alternative ways to derive canonical correlations. In addition to using Pearson's correlation to measure the strength of brain-behavior coupling in eFigure 3, we also used linear mixed-effect models with subject-specific intercepts to measure the coupling strength accounting for the repeated measures (multiple visits) per participant. Specifically, for each component identified on the training folds, the mixed-effect model regressed the functional canonical variables from the neuropsychological variables in the testing fold. The distribution of regression slope associated the first four components in the executive control system and the first three components in socioemotional system remained significant according to a permutation test (p<0.0001, eFigure 4).

Next, we repeated the CCA analysis based on two alternative procedures of processing rs-fMRI connectivity measurements. The first alternative did not average the connectivity between bilateral regions but directly applied PCA to the full connectivity matrix (of size 109 x 109). eFigure 5 suggests that this procedure resulted in the same number of significant components as in eFigure 3, but generally with smaller canonical correlation values. The second alternative used the bilaterally averaged connectivity measures without PCA reduction, which resulted in fewer significant components with weaker canonical correlations. These results collectively suggest that applying PCA to the bilaterally averaged connectivity measures could maximally reveal brain-behavior association patterns.

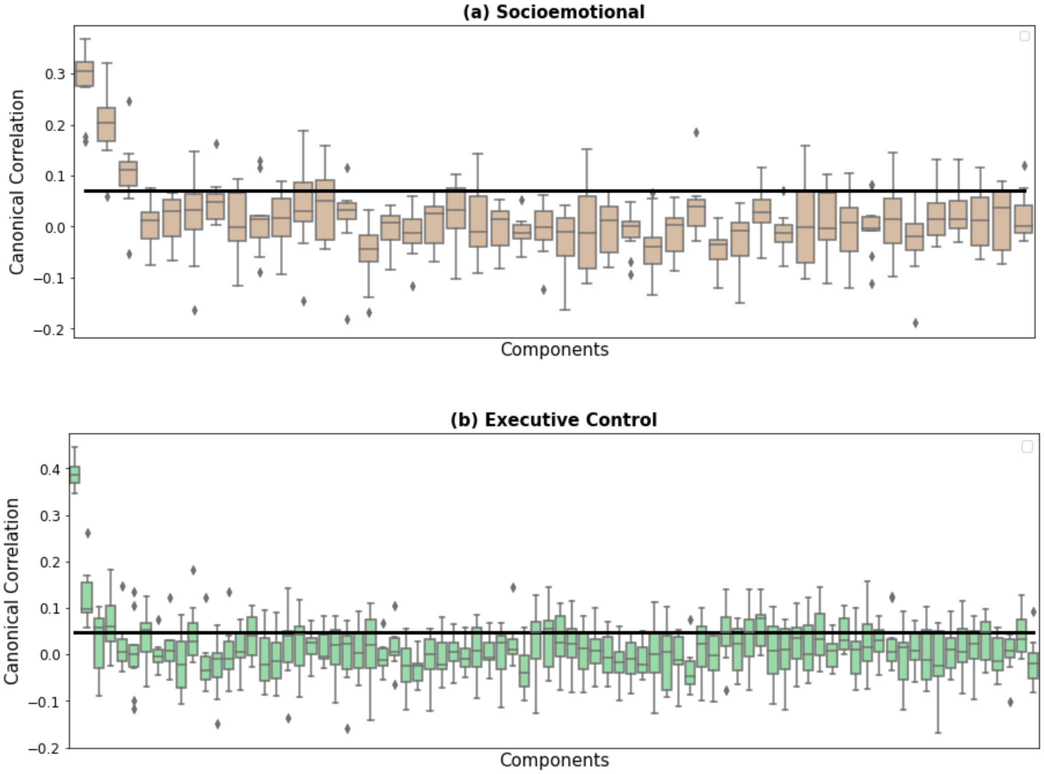

eFigure 4. Distribution of Regression Slope of Linear Mixed-Effects Models. Distribution of regression slope of linear mixed-effect (LME, with subject-specific intercepts) models that regressed the neuropsychological canonical variables from functional canonical variables in each canonical component. Both functional and neuropsychological canonical variables underwent z-score normalization, so that the resulting regression slopes of LME were

© 2025 Zhao Q et al. *JAMA Network Open*.

commensurate across components. Black lines indicate significance threshold at p=0.0001 based on permutation tests.

© 2025 Zhao Q et al. *JAMA Network Open*.

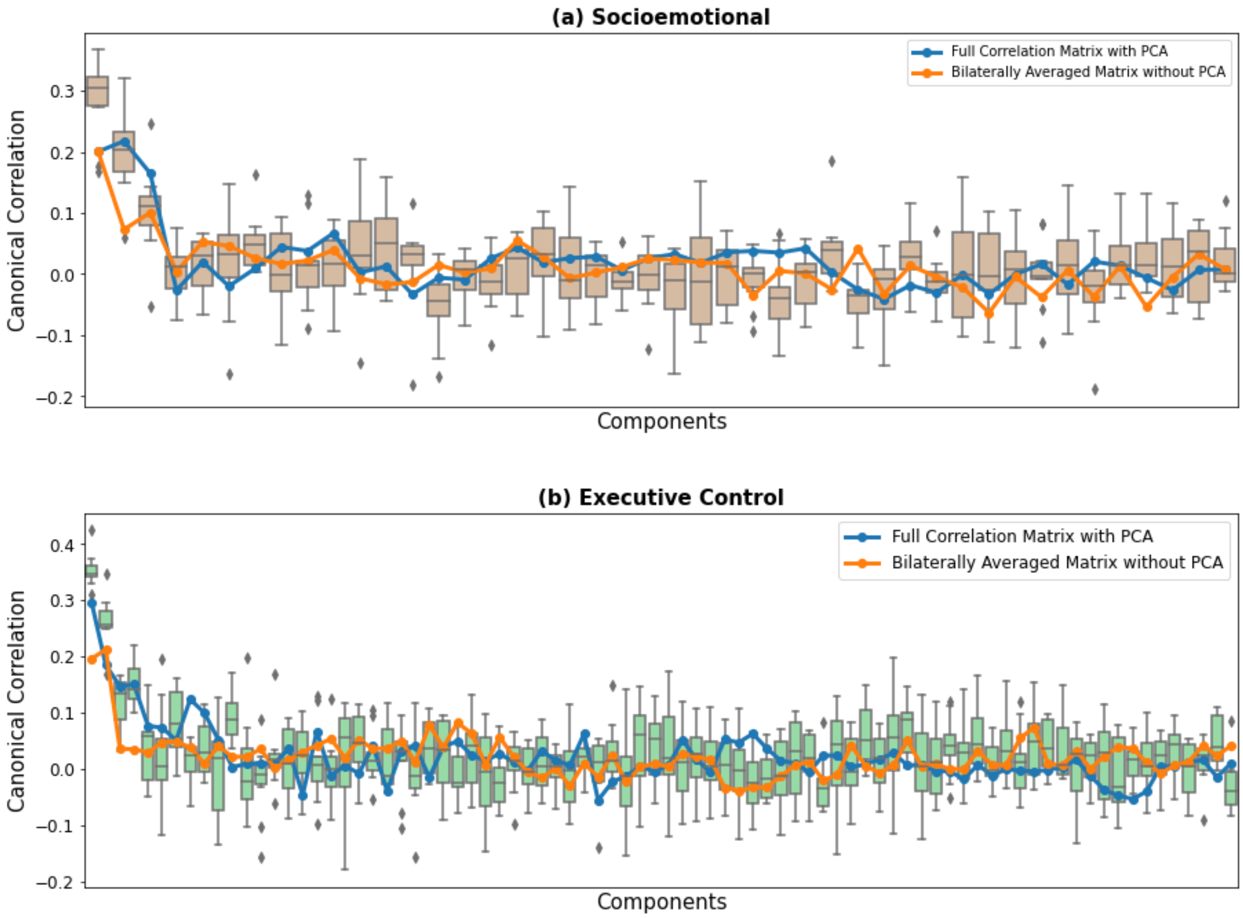

eFigure 5. Distribution of Canonical Correlation Values Across Processing Methods of Resting-State Functional Data. Comparison of the distribution of canonical correlation values across different processing methods of resting-state functional data: Boxplots correspond to the distribution of canonical correlation values in eFigure 3 (PCA on the bilaterally averaged connectivity measures). The blue curve corresponds to the median canonical correlation based on applying PCA to the full correlation matrix. The orange curve corresponds to the median correlation of using bilaterally averaged matrix without PCA reduction.

© 2025 Zhao Q et al. *JAMA Network Open*.

eTable 3. Top 5 Functional Connectivities (Between Pairs of Regions) With Highest Canonical Loadings for Each Significant Canonical Component in eFigure 3

Component	Socioemotional	Executive Control
1	Caudate <> Thalamus<br>Putamen <> Thalamus<br>Paracentral_Lobule <> Thalamus<br>Rolandic_Oper <> Putamen<br>Insula <> Putamen	Cerebelum_8 <> Cerebelum_10<br>Paracentral_Lobule <> Thalamus<br>Parietal_Sup <> Cerebelum_10<br>Frontal_Inf_Oper <> Insula<br>Cingulum_Ant <> Thalamus
2	Temporal_Pole_Sup <> Temporal_Pole_Mid<br>Frontal_Sup_Medial <> Temporal_Pole_Sup<br>Frontal_Sup <> Hippocampus<br>Cingulum_Mid <> Angular<br>Insula <> Cerebelum_Crus2	Frontal_Sup_Medial <> Temporal_Pole_Sup<br>Frontal_Sup_Medial <> Temporal_Pole_Mid<br>ParaHippocampal <> Occipital_Sup<br>Cerebelum_Crus1 <> Cerebelum_4_5<br>Precentral <> Occipital_Inf
3	Cerebelum_9 <> Cerebelum_10<br>Frontal_Mid <> Frontal_Inf_Tri<br>Parietal_Sup <> Temporal_Pole_Sup<br>Precentral <> Paracentral_Lobule<br>Frontal_Mid <> Frontal_Inf_Oper	Precentral <> Postcentral<br>Cingulum_Mid <> Calcarine<br>Cingulum_Mid <> Cuneus<br>Angular <> Temporal_Mid<br>Angular <> Precuneus
4		Frontal_Med_Orb <> Temporal_Mid<br>Frontal_Sup <> Frontal_Inf_Tri<br>Insula <> Cerebelum_3<br>Cerebelum_Crus2 <> Cerebelum_10<br>Occipital_Sup <> Temporal_Inf

Alternative Mixed-Effect Models: We constructed two different statistical models to test the reproducibility of the trajectory difference between heavy and non-heavy drinkers observed in Fig. 2&4 of the main text. First, for those 4 components showing significant alcohol effects (main text Fig. 2a-c, 4b), we repeated the mixed-effect regression analysis by replacing the brain-behavior score (the average of functional canonical variable and the neuropsychological canonical variable) with either the functional canonical variable alone or the neuropsychological canonical variable alone. eTable 4 shows that all significant effects persisted, suggesting the alcohol effects exist in both functional connectivity and neuropsychological measures.

Next, we redefined the two drinking groups by separating those who remained no-to-low drinking (Cahalan score = 0) throughout the study from those who initiated moderate-to-heavy drinking (Cahalan score >= 1) during the study. Rerunning the mixed-effect models on these two new drinking groups resulted in the same results (eTable 5).

Third, we repeated the mixed-effect models by adding race, site, and socioeconomic status as covariates. As expected, none of these additional covariates had significant effects on the brain-behavior scores as their influences were already removed from original functional connectivity and neuropsychological measures before the CCA analysis. Therefore, all significant alcohol effects persisted (eTable 6).

Lastly, we noticed sex always had a significant effect on the component scores in all mixed-effects models. Given that there was a significant sex difference (main text, Table 1) between the non-heavy drinkers and heavy drinkers, we repeated the mixed-

© 2025 Zhao Q et al. *JAMA Network Open*.

effect models on a subset of the 633 participants, where we randomly omitted 85 non-heavy-drinking females, so that the sex ratio was matched between the two drinking groups. Repeating this procedure 100 times, eFigure 6a indicates the alcohol effects could be consistently identified on the sex-matched samples. Following a similar procedure, we also randomly omitted 157 participants from the non-heavy drinking group so that the two groups were exactly matched by race. eFigure 6b indicates the alcohol effects could be consistently identified on the race-matched samples.

eTable 4. *P* Values of the Group Differences Between Heavy Drinkers and Nonheavy Drinkers Based on Different Regression Targets. *P* values (Bonferroni corrected for 7 components) of the group difference between heavy drinkers (during heavy-drinking visits) and non-heavy drinkers based on different regression targets (functional canonical variable, neuropsychological canonical variable, or the average brain-behavior score) in the LME model.

	Executive Control			Socioemotional		
	Brain-Behavior Score	Functional Canonical Variable	Neuropsychological Canonical Variable	Brain-Behavior Score	Functional Canonical Variable	Neuropsychological Canonical Variable
Component 1	1	0.710	1	<0.001	<0.001	<0.001
Component 2	<0.001	0.002	0.002	<0.001	<0.001	<0.001
Component 3	1	1	1	<0.001	<0.001	<0.001
Component 4	0.546	0.726	0.499			

eTable 5. The Same LME Models in eTable 4 Applied to Different Groupings of Participants Into Drinkers (Cahalan Score > 0) and Nondrinkers (Cahalan Score = 0)

	Executive Control			Socioemotional		
	Brain-Behavior Score	Functional Canonical Variable	Neuropsychological Canonical Variable	Brain-Behavior Score	Functional Canonical Variable	Neuropsychological Canonical Variable
Component 1	0.405	0.180	1	<0.001	<0.001	<0.001
Component 2	<0.001	0.003	<0.001	<0.001	<0.001	<0.001
Component 3	1	1	1	<0.001	0.028	<0.001
Component 4	0.324	0.366	0.174			

eTable 6. Results of LME With Additional Covariates of Race, Site, and Socioeconomic Status

	Executive Control			Socioemotional		
	Brain-Behavior Score	Functional Canonical Variable	Neuropsychological Canonical Variable	Brain-Behavior Score	Functional Canonical Variable	Neuropsychological Canonical Variable
Component 1	0.955	0.508	1	<0.001	<0.001	<0.001
Component 2	<0.001	<0.001	<0.001	<0.001	<0.001	<0.001
Component 3	1	1	1	<0.001	<0.001	<0.001
Component 4	0.515	0.755	0.435			

© 2025 Zhao Q et al. *JAMA Network Open*.

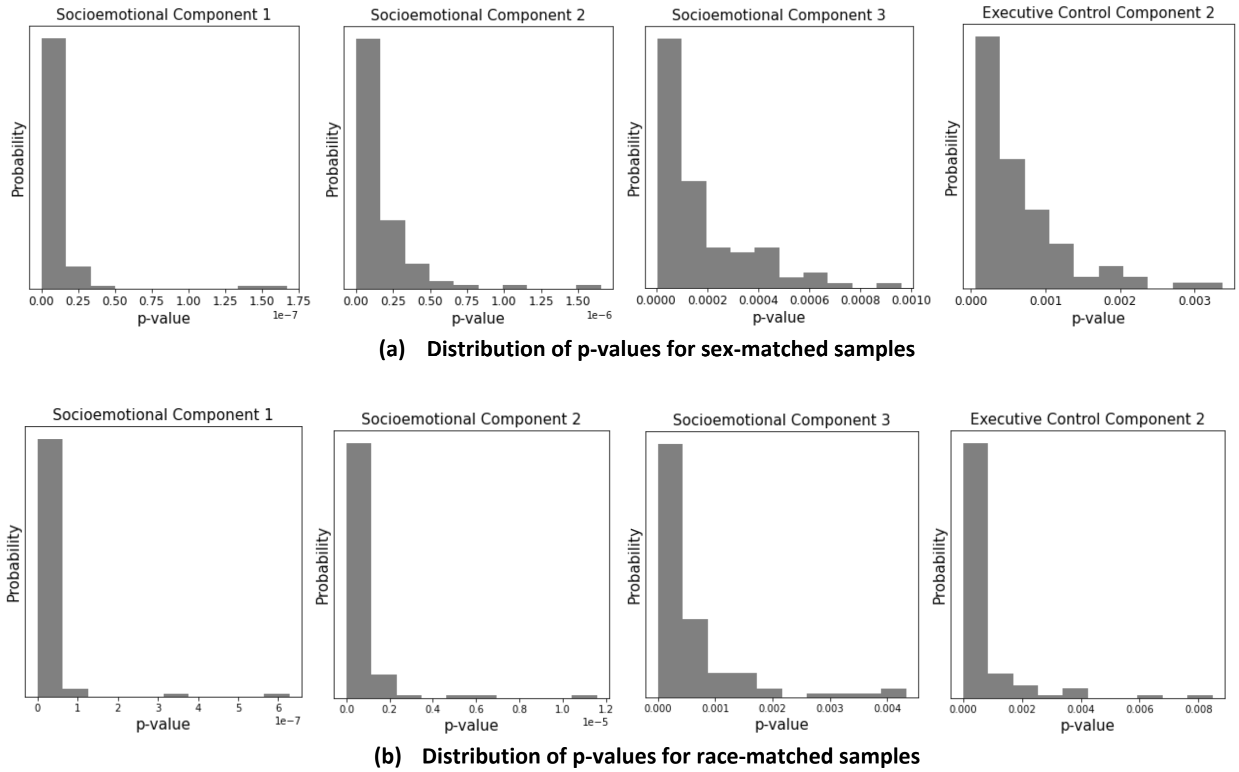

eFigure 6. Distribution of *P* Values (Uncorrected) Associated With the Alcohol Effects When Running Mixed-Effects Models on Randomly Sampled Cohorts: (a) sex-matched cohorts; (b) and race-matched cohorts.

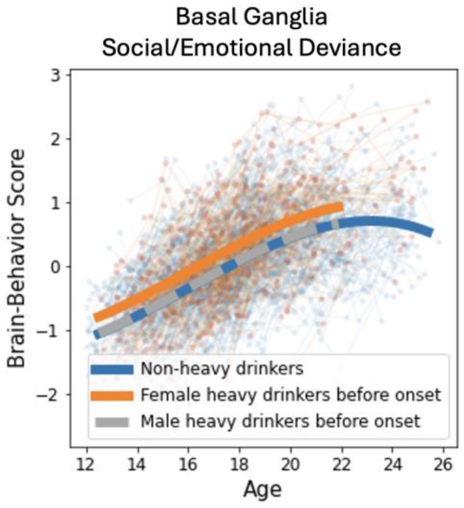

eFigure 7. Significant Sex-Alcohol Interaction in the First Socioemotional Component. In the 1st socioemotional component, a significant sex-alcohol interaction was detected (p=0.014, Bonferroni corrected for the number of components with significant alcohol effects). Only female heavy drinkers showed elevated brain-behavior scores before heavy drinking onset compared to the norm.

© 2025 Zhao Q et al. *JAMA Network Open*.

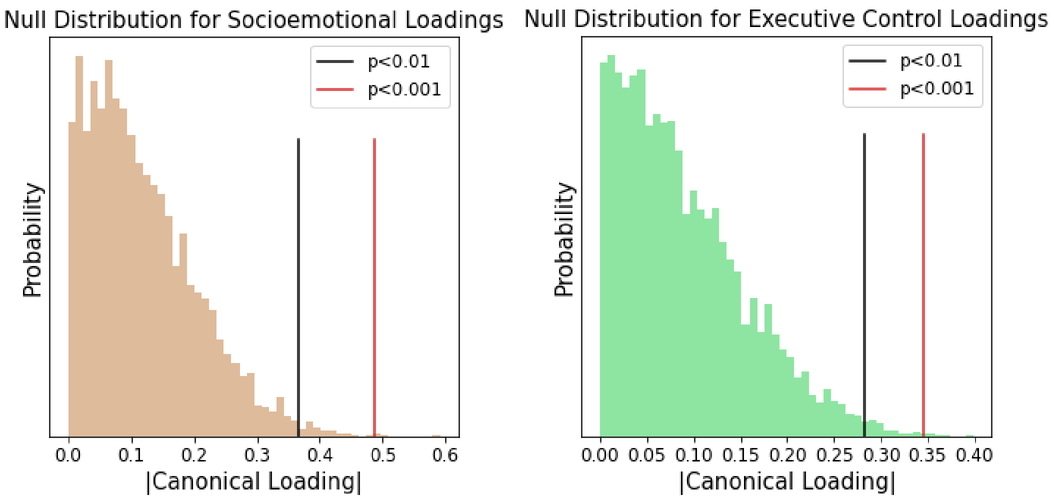

eFigure 8. Null Distribution of Canonical Loadings for Individual Neuropsychological Measures of the Socioemotional and Executive Control Systems

© 2025 Zhao Q et al. *JAMA Network Open*.

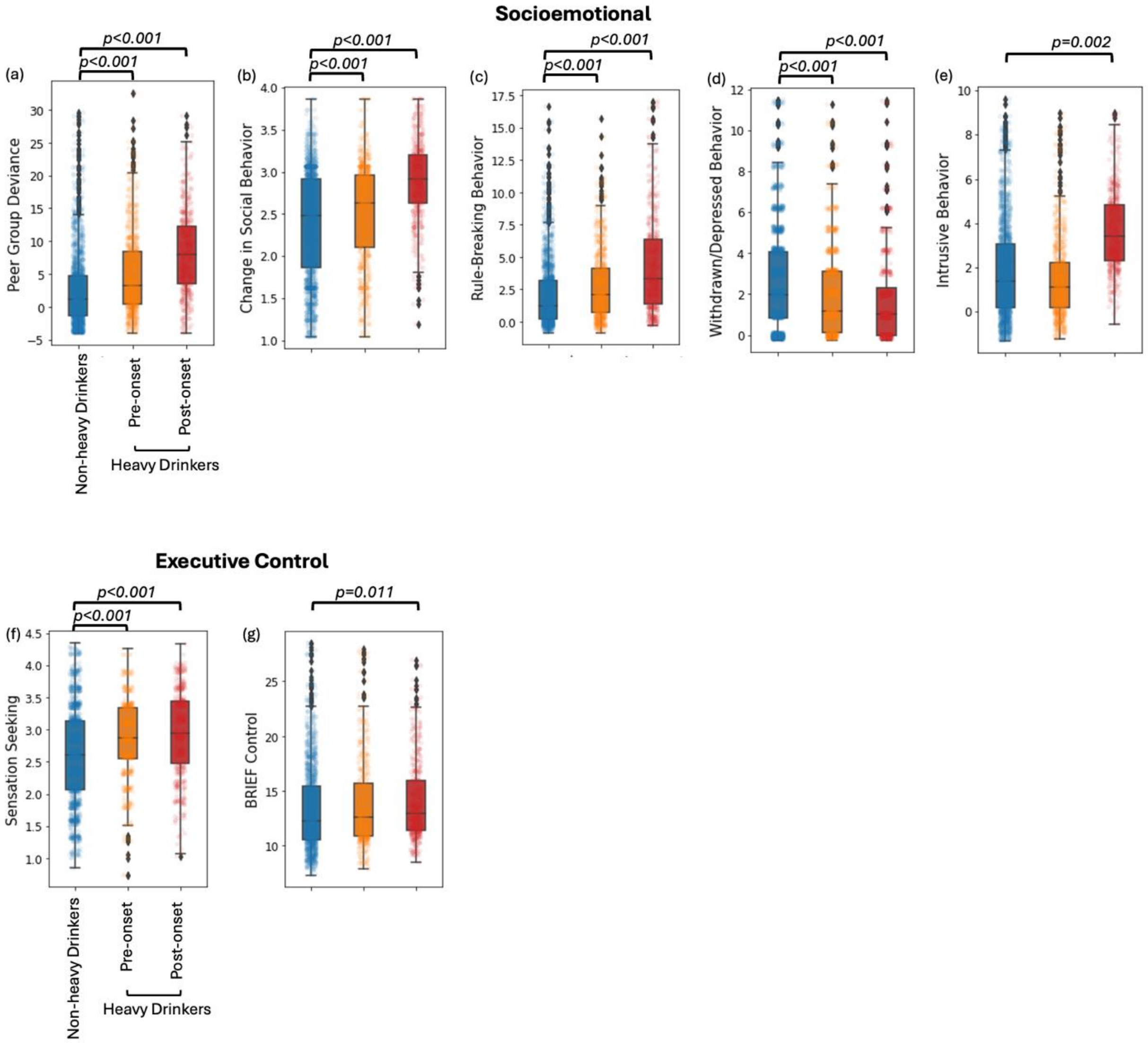

eFigure 9. Distribution of Top Neuropsychological Measures Defining the Canonical Components That Showed Significant Alcohol Effects (main text Fig. 2a,2b,2c,4b). Covariates effects of age, sex, race, and socioeconomic status were regressed out from the measures. Blue boxes correspond to measures of non-heavy drinkers. Orange and red boxes correspond to measures of heavy drinkers before and after drinking onset.

© 2025 Zhao Q et al. *JAMA Network Open*.

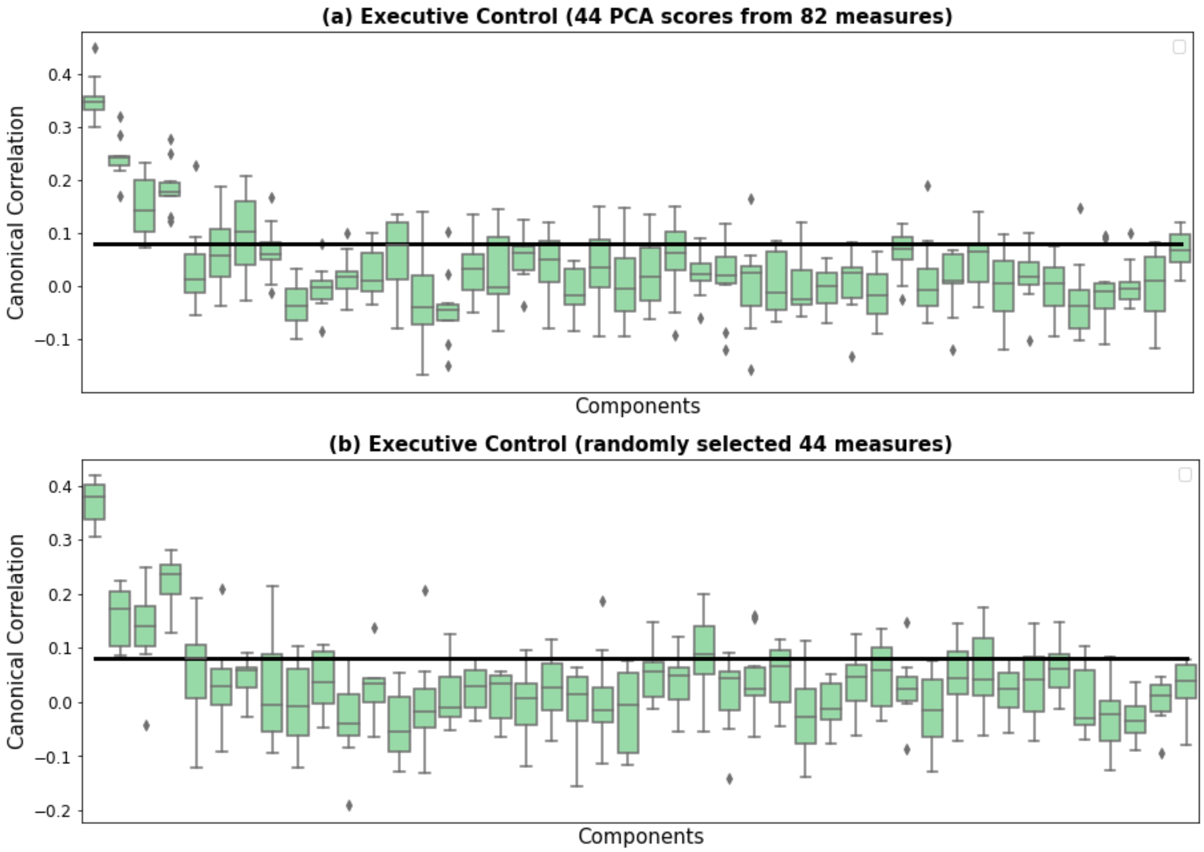

eFigure 10. Distributions of Canonical Correlations. Distributions of canonical correlation between functional connectivity and (a) 44 PCA scores derived from 82 executive control measures (b) 44 randomly selected measures from the 82 measures. The black line indicates the null threshold at the p=0.0001 level.

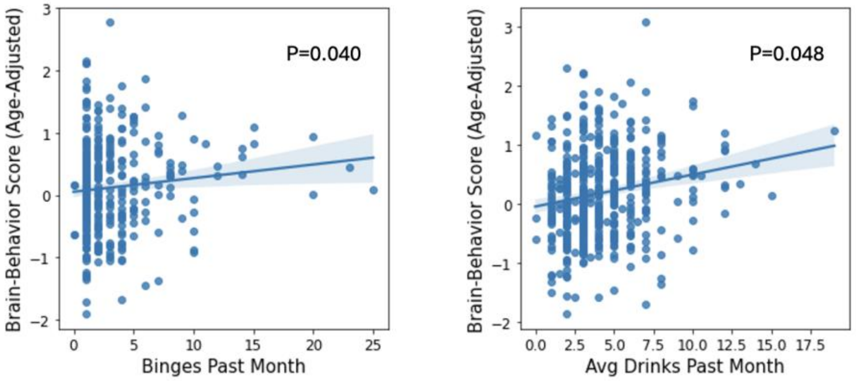

eFigure 11. Brain-Behavior Score of the Second Component of the Executive Control System. The brain-behavior score of the 2nd component of the executive control system significantly correlated with past month alcohol consumption among heavy drinkers after onset. p-values were Bonferroni corrected for 8 tests.

© 2025 Zhao Q et al. *JAMA Network Open*.

Relationship between components of the two systems. To examine to what extent the canonical components from the executive control system coactivate with the components from the socioemotional system, we correlated the brain-behavioral scores of the 633 participants between each pair of executive control component and socioemotional component. Supplement eFigure 12 reveals that 6 pairs of canonical components show significant correlation at $p<0.01$. However, the effect size of those regression models (R2) was generally low. On average, the brain-behavior scores of an executive control component only explained about 2% variance in the brain-behavior scores of a socioemotional component. These results indicate substantial segregation between the two neural systems.

eReferences

1. Brown, S.A., et al., *The National Consortium on Alcohol and NeuroDevelopment in Adolescence (NCANDA): A Multisite Study of Adolescent Development and Substance Use.* J Stud Alcohol Drugs, 2015. **76**(6): p. 895-908.
2. Pfefferbaum, A., et al., *Altered Brain Developmental Trajectories in Adolescents After Initiating Drinking.* Am J Psychiatry, 2018. **175**(4): p. 370-380.
3. Cahalan, D., I.H. Cisin, and H.M. Crossley, *American drinking practices: A national study of drinking behavior and attitudes. Monographs of the Rutgers Center of Alcohol Studies*, 1969. **6**(260).
4. Wang, H.T., et al., *Finding the needle in a high-dimensional haystack: Canonical correlation analysis for neuroscientists.* Neuroimage, 2020. **216**: p. 116745.
5. Brown, S.A., B.A. Christiansen, and M.S. Goldman, *The Alcohol Expectancy Questionnaire: an instrument for the assessment of adolescent and adult alcohol expectancies.* J Stud Alcohol, 1987. **48**(5): p. 483-91.
6. Achenbach, T.M., et al., *Multicultural assessment of child and adolescent psychopathology with ASEBA and SDQ instruments: research findings, applications, and future directions.* J Child Psychol Psychiatry, 2008. **49**(3): p. 251-75.
7. Radloff, L.S., *CES-D scale: A self report depression scale for research in the general populations.* Applied Psychological Measurement, 1977. **1**: p. 385-401
8. Miley, A., G. Kecklund, and T. Åkerstedt, *Comparing two versions of the Karolinska Sleepiness Scale (KSS).* Sleep Biol Rhythms, 2016. **14**(3): p. 257-260.
9. Bernstein, D.P., et al., *Initial reliability and validity of a new retrospective measure of child abuse and neglect.* Am J Psychiatry, 1994. **151**(8): p. 1132-6.
10. Connor-Smith, J.K., et al., *Responses to stress in adolescence: measurement of coping and involuntary stress responses.* J Consult Clin Psychol, 2000. **68**(6): p. 976-92.
11. Gilreath, T.D., et al., *Substance use among military-connected youth: the California Healthy Kids Survey.* Am J Prev Med, 2013. **44**(2): p. 150-3.
12. Nunes, A., et al., *Short Scales for the Assessment of Personality Traits: Development and Validation of the Portuguese Ten-Item Personality Inventory (TIPI).* Front Psychol, 2018. **9**: p. 461.

© 2025 Zhao Q et al. *JAMA Network Open*.

13. Gioia, G.A., et al., *Confirmatory factor analysis of the Behavior Rating Inventory of Executive Function (BRIEF) in a clinical sample.* Child Neuropsychol, 2002. **8**(4): p. 249-57.
14. Gur, R.C., et al., *A cognitive neuroscience-based computerized battery for efficient measurement of individual differences: standardization and initial construct validation.* J Neurosci Methods, 2010. **187**(2): p. 254-62.
15. Stanger, C., et al., *Delay discounting predicts adolescent substance abuse treatment outcome.* Exp Clin Psychopharmacol, 2012. **20**(3): p. 205-12.
16. Schmidt, S.L., et al., *Influences of handedness and gender on the grooved pegboard test.* Brain Cogn, 2000. **44**(3): p. 445-54.
17. Shin, M.S., et al., *Clinical and empirical applications of the Rey-Osterrieth Complex Figure Test.* Nat Protoc, 2006. **1**(2): p. 892-9.
18. Tombaugh, T.N., *A comprehensive review of the Paced Auditory Serial Addition Test (PASAT).* Arch Clin Neuropsychol, 2006. **21**(1): p. 53-76.
19. Scarpina, F. and S. Tagini, *The Stroop Color and Word Test.* Front Psychol, 2017. **8**: p. 557.
20. Cyders, M.A., et al., *Examination of a short English version of the UPPS-P Impulsive Behavior Scale.* Addict Behav, 2014. **39**(9): p. 1372-6.

© 2025 Zhao Q et al. *JAMA Network Open*.
