# Supplementary material for: Socioemotional and Executive Control Mismatch in Adolescence and Risks for Initiating Drinking
Source: JAMA Netw Open. 2025 Sep 12;8(9):e2531378. doi: 10.1001/jamanetworkopen.2025.31378 (PMC12432642; doi:10.1001/jamanetworkopen.2025.31378)
Supplement: Supplement 2. — Data Sharing Statement [file jamanetwopen-e2531378-s002.pdf]

Data Sharing Statement

Zhao. Socioemotional and Executive Control Mismatch in Adolescence and Risks for Initiating Drinking. *JAMA Netw Open*. Published September 12, 2025.
doi:10.1001/jamanetworkopen.2025.31378

Data
Data available: Yes
Data types: Deidentified participant data
How to access data: http://www.ncanda.org/datasharing.php
When available: With publication

Supporting Documents
Document types: Statistical/analytic code
How to access documents: Upon request: qiz4006@med.cornell.edu
When available: With publication

Additional Information
Who can access the data: researchers whose proposed use of the data has been approved
Types of analyses: for any purpose
Mechanisms of data availability: http://www.ncanda.org/datasharing.php
